# Haloperoxidase Mediated Quorum Quenching by *Nitzschia cf pellucida*: Study of the Metabolization of *N*-Acyl Homoserine Lactones by a Benthic Diatom

**DOI:** 10.3390/md12010352

**Published:** 2014-01-17

**Authors:** Michail Syrpas, Ewout Ruysbergh, Lander Blommaert, Bart Vanelslander, Koen Sabbe, Wim Vyverman, Norbert De Kimpe, Sven Mangelinckx

**Affiliations:** 1 Department of Sustainable Organic Chemistry and Technology, Faculty of Bioscience Engineering, Ghent University, Coupure Links 653, Ghent B-9000, Belgium; E-Mails: Michail.Syrpas@UGent.be (M.S.); Ewout.Ruysbergh@UGent.be (E.R.); Norbert.DeKimpe@UGent.be (N.D.K.); 2 Laboratory of Protistology and Aquatic Ecology, Department of Biology, Ghent University, Krijgslaan 281-S8, Ghent B-9000, Belgium; E-Mails: Lander.Blommaert@UGent.be (L.B.); Bart.Vanelslander@gmail.com (B.V.); Koen.Sabbe@UGent.be (K.S.)

**Keywords:** quorum sensing, haloperoxidase, degradation pathway, AHL, reference compounds, diatom-bacteria interactions

## Abstract

Diatoms are known to produce a variety of halogenated compounds, which were recently shown to have a role in allelopathic interactions between competing species. The production of these compounds is linked to haloperoxidase activity. This research, has shown that this system may also be involved in diatom-bacteria interactions via the H_2_O_2_ dependent inactivation of a type of quorum sensing (QS) molecule, *i.e.*, *N*-β-ketoacylated homoserine lactones (AHLs), by a natural haloperoxidase system from the benthic diatom *Nitzschia cf pellucida*. The AHL degradation pathway towards corresponding halogenated derivatives was elucidated via HPLC-MS analysis and the synthesis of a broad series of novel halogenated AHL analogues as reference compounds. Furthermore, their biological activity as quorum sensing modulators was directly compared and evaluated against a series of naturally occurring β-keto-AHLs. It has been demonstrated that the loss of the QS activity results from the final cleavage of the halogenated *N*-acyl chain of the signal molecules.

## 1. Introduction

Algae are known as an important source of bioactive compounds with potential agrochemical and pharmaceutical applications [1]. Some of these bioactive metabolites include halogenated compounds [2]. These types of compound often demonstrate interesting biological properties including antifungal, anti-inflammatory, antifeedant, cytotoxic, insecticidal, and antibacterial activity [3]. Biohalogenation in marine organisms usually occurs via the action of haloperoxidases [4], which are enzymes that mediate the oxidation of halides by hydrogen peroxide via various mechanisms [5]. Until now, screening for bioactive halogenated compounds in marine environments has mainly been focused on macroalgae rather than on microalgae [2]. Research on diatoms, one of the most common type of marine microalgae, is even less widespread.

Many bacteria use signal molecules, *i.e.*, autoinducers, to control the expression of specific genes important for phenotypes, such as biofilm formation, bioluminescence, virulence expression, motility, conjugation, and symbiosis and this in a population density dependent manner [6]. This interbacterial signaling is widely known as quorum sensing (QS). *N*-Acylated homoserine lactones (AHLs) represent a major class of these signal molecules, and are commonly produced by Gram negative bacteria [7]. The QS system in these bacteria usually consists of two components: a LuxI homologue that synthesizes the AHL autoinducer, and a LuxR homologue which is an autoinducer-dependent transcriptional activator [7]. Different bacterial species have been shown to produce different AHLs [8]. The basic structure of AHLs consists of a homoserine lactone ring adjoined with an *N*-acyl chain. The acyl chain can vary in length from four to 14 carbon atoms, as can the degree of saturation and the chain may or may not contain an oxo- or hydroxy-group at the 3-carbon position [9]. A wide variety of AHLs, including various saturated and β-keto-AHLs, are found in marine stromatolite mats but also in intertidal surfaces colonized by algae and in intertidal rock pools [10,11]. AHLs are also known to modulate the settlement rate of green alga *Ulva intestinalis* [12]*.* The AHL producing *Alphaproteobacteria*, which are dominant in benthic diatom biofilms, were shown to have an influence on the diatom growth and the release of extracellular polymeric substances [13]. Across different kingdoms these kinds of interactions may exert wider ecological effects [14].

In aquatic environments, bacteria interact in various ways with other organisms as pathogens, symbionts or competitors in the search for resources [14,15,16]. Many marine organisms have developed defense or deterrence mechanisms, which are often involved in the production of small diffusible signal molecules that can interfere with the QS systems [17,18]. A well-studied example is the production of halogenated furanones by the red alga *Delisea pulchra* [19,20]. In addition, QS antagonistic activity has been demonstrated in algae from *Caulerpaceae* (green algae), *Rhodomelaceae* and *Galaxauraceae* families (red algae) [21], red macroalga *Asparagopsis taxiformis* [22], green microalga *Chlamydomonas reinhardtii* [23], freshwater green microalga *Chlorella sacharophilla* [24], gorgonian corals from the Caribbean reefs [25] and the marine sponge *Luffariella variabilis* [26,27]. Furthermore, another way of QS disruption in nature occurs via the action of enzymes. Enzymatic degradation of QS signal molecules by acylases and lactonases of bacterial origin has been demonstrated [28]. In another pathway, haloperoxidases from *Delisea pulchra* and *Laminaria digitata* were shown to mediate the deactivation of β-keto-AHLs by electrophilic halogenation with bromine [29,30]. Several diatoms are linked with the release of reactive bromine and iodine which is influenced by temperature, H_2_O_2_ concentration, bromide (and iodide) concentration, light, and pH [31]. The release leads to the formation of a wide range of halocarbons, formed via rapid and aspecific reactions of these reactive halogen intermediates with many organic molecules. Furthermore, this event plays a substantial role in oceanic bromoform production via the haloform reaction [32]. Interestingly, the production of volatile halocarbons by several *Nitzschia* species has been linked to haloperoxidase activity [33,34]. Nevertheless, not much is known about QS antagonistic compounds derived from diatoms. To the best of our knowledge there is no data which demonstrates the release of reactive bromine (or iodine) from diatoms as a possible defense mechanism against bacterial fouling.

In the present study, it was envisioned that the natural haloperoxidase system from the benthic diatom *N. cf pellucida*, besides allelopathic interactions between competing species, may also be involved in the deactivation of bacterial QS molecules. Therefore, the goal of this study was to analyze the effect of haloperoxidase activity by the diatom *N. cf pellucida* on bacterial QS. During this research, the degradation pathway of the QS molecules was elucidated and a variety of halogenated AHL degradation derivatives were synthesized and evaluated for their ability to either induce or inhibit QS activity.

## 2. Results and Discussion

### 2.1. *Nitzschia cf pellucida* Deactivation of β-Keto-AHLs

**Figure 1:**
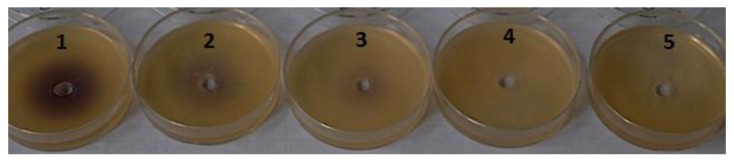
Loss of quorum sensing (QS) activity of *N*-3-oxohexanoyl homoserine lactone (OHHL) in the *Chromobacterium violaceum* CV026 bioassay monitored over time. (*N. cf pellucida* + H_2_O_2_ + OHHL treatment). Where, 1: *t* = 0 min; 2: *t* = 30 min; 3: *t* = 60 min; 4: *t* = 120 min; 5: *t* = 180 min.

A first series of experiments was performed in order to determine whether the haloperoxidase system within *Nitzschia cf pellucida* is capable of mediating the deactivation of AHLs. In a previous study, the presence of a haloperoxidase in this benthic diatom was detected via the phenol red assay [33]. In the present study, haloperoxidase activity was verified via the formation of bromophenol blue after addition of phenol red and H_2_O_2_ (to ensure sufficient production of reactive halogen intermediates [29,30]) to the cultures (data not shown) [33]. Furthermore, the activity of *N*-3-oxohexanoyl homoserine lactone (OHHL) and *N*-hexanoyl homoserine lactone (HHL) was monitored over time with the *Chromobacterium violaceum* CV026 bioassay in the absence or presence of *N. cf pellucida* (Table 1). These compounds were selected, because OHHL is a known biofilm enhancer. For the control sample (synthetic seawater + H_2_O_2_ + OHHL) and the sample in which the *N. cf pellucida* culture was only supplemented with OHHL, the QS activity was retained throughout the monitoring period. The decrease in QS activity that was observed for these samples after 180 min of incubation is most probably due to pH-dependent AHL lactonolysis [35]. The addition of synthetic homoserine lactone bearing a β-keto moiety (OHHL) in combination with H_2_O_2_ to a *N. cf pellucida* culture resulted in the deactivation of this type of AHL which is demonstrated in Figure 1. This is in accordance with previous studies in which natural haloperoxidase systems of *Laminaria digitata* and *Delisea pulchra* mediated the deactivation of these AHLs in *C. violaceum* CV026 [30] and *A. tumefaciens* NT1 bioassays [29]. Activity of β-keto-AHL was lost after 60 min in cultures in which H_2_O_2_ was added. Not surprisingly, HHL which does not contain a β-keto moiety, was not affected and retained QS activity. In a next step, in order to verify whether the deactivation of the OHHL was due to haloperoxidase activity, two additional experiments were performed. In the first experiment, catalase was added to an active *N. cf pellucida* culture. Catalase is an enzyme that catalyzes the decomposition of hydrogen peroxide, which occurs in algal cells due to the Mehler reaction [36], to water and oxygen. It was expected that in the absence of hydrogen peroxide, the haloperoxidase would not be able to mediate the oxidation of halides and subsequently the halogenation of OHHL. This assumption was found to be true as can be seen from the final entry in Table 1. Similar to the sample containing the culture and OHHL, the QS stimulating activity was retained for the culture where catalase and H_2_O_2_ were added, which demonstrates that the deactivation of β-keto-AHLs is H_2_O_2_-dependent.

**Table 1:** Effect of *N. cf pellucida* haloperoxidase on the quorum sensing (QS) activity of signal molecules (*N*-hexanoyl homoserine lactone (HHL) and *N*-3-oxohexanoyl homoserine lactone (OHHL)), assessed by the *Chromobacterium violaceum* CV026 bioassay (+ + + strong QS activity, − no QS activity).

Sample	0 min	30 min	60 min	120 min	180 min
Synthetic seawater + H_2_O_2_ + OHHL	+ + +	+ + +	+ + +	+ +	+
*N. cf pellucida*	−	−	−	−	−
*N. cf pellucida* + OHHL	+ + +	+ + +	+ + +	+ +	+
*N. cf pellucida* + H_2_O_2_ + OHHL	+ + +	+ +	+	−	−
*N. cf pellucida* + HHL	+ + +	+ + +	+ + +	+ +	+ +
*N. cf pellucida* + H_2_O_2_ + HHL	+ + +	+ + +	+ + +	+ +	+ +
*N. cf pellucida* + H_2_O_2_ + OHHL + Catalase	+ + +	+ + +	+ + +	+ +	+

The second experiment was performed in order to evaluate the possibility that cyanogen bromide (BrCN) could react with the signal molecules and cause the deactivation (Table 2). BrCN is one of the main and most active halogenated metabolites previously identified in *N. cf pellucida* extracts [33]. Two different concentrations (2 and 4 μM) within the range of BrCN levels produced by *N. cf pellucida* were evaluated. From these experiments, it could be seen that the addition of BrCN did not result in the deactivation of the signal molecules. Moreover, no inhibition of bacterial growth was observed for the control samples, which is in accordance with a previous study [33].

**Table 2:** QS activity of *N*-Acylated homoserine lactones (AHLs) in the *Chromobacterium violaceum* CV026 bioassay in the presence of two different concentrations of cyanogen bromide (BrCN) (+ + + strong QS activity, − no QS activity).

Sample	Activity
2 μM BrCN	−
4 μM BrCN	−
2 μM BrCN + HHL	+ + +
4 μM BrCN + HHL	+ + +
2 μM BrCN + OHHL	+ +
4 μM BrCN + OHHL	+ +

### 2.2. Degradation Pathway of β-Keto-AHLs

**Scheme 1:**
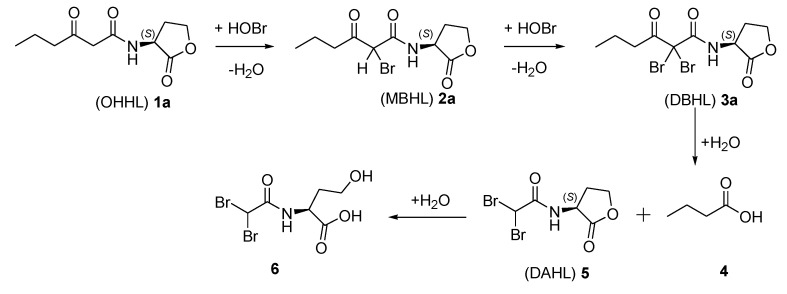
Degradation pathway of *N*-3-oxohexanoyl homoserine lactone (OHHL) (**1a**). Scheme adopted from [37].

The formation of halogenated compounds by a haloperoxidase system can be elaborated either via direct binding of the substrate to the active site or via the release of hypohalous acids from the active site. The latter can occur via bromide, chloride or iodide oxidation by hydrogen peroxide, which has an effect on electrophilic halogenating reactions. The suggested degradation pathway of *N-*β-keto-acylated homoserine lactone bacterial signaling molecules is presented in Scheme 1 [37].

The degradation pathway of β-keto-AHL bacterial signaling molecules by the natural haloperoxidase system of *N. cf pellucida* was investigated using a reversed-phase HPLC separation method developed to detect the parent lactones and the occurring halogenated products. Structure elucidation of the anticipated reaction products was achieved by photodiode array UV spectroscopy coupled with mass spectrometry. The chromatographic analysis of the degradation of OHHL (**1a**, Scheme 1) revealed the formation of the brominated products **2a**, **3a** and **5** (Table 3). OHHL yielded the (M + H)^+^ ion with the anticipated *m/z* value of 214. The peaks eluting at a later stage were assumed to be the monobrominated OHHL (MBHL, **2a**) and the dibrominated OHHL (DBHL, **3a**). Indeed, mass spectrometric analysis yielded a pseudomolecular ion with *m/z* value of 292/294 that can be attributed to MBHL (**2a**) (retention time 15.7 min). The 1:1 isotope peak pattern further indicated the addition of one bromine atom to OHHL. The *m/z* values of 370/372/374 (retention time 19.3 min) showed a 1:2:1 isotope peak pattern which is consistent with the DBHL (**3a**), formed by the introduction of two bromine atoms. Cleavage of the halogenated *N*-acyl chain resulted subsequently in the formation of *N*-(α,α-dibromoacetyl) homoserine lactone (DAHL, **5**). For the latter compound **5** a pseudomolecular ion with *m/z* value of 300/302/304 was observed, again with the characteristic 1:2:1 isotope peak pattern. Finally, structure elucidation of these compounds was further verified via the preparation of synthetic standards **1**, **2**, **3** and **5** as reference compounds (see Section 2.3). To the best of our knowledge this is the first time that the suggested degradation pathway has been verified by the use of reference compounds.

**Table 3:** Degradation of *N*-3-oxohexanoyl homoserine lactone (OHHL) (**1a**) analyzed by HPLC-MS.

Compound	(M + H)^+^	R.T. (min) ^a^	Relative Abundance (%) at 220 nm			
			30 min	60 min	120 min	180 min
(OHHL) **1a**	214	5.2	37	29	25	<3
(MBHL) **2a**	292	15.7	21	24	27	27
(DBHL) **3a**	370	19.3	42	47	48	63
(DAHL) **5**	300	4.1	0	0	0	7

^a^ Retention time (R.T.) corresponds with the R.T. of synthetically prepared reference compounds.

### 2.3. Synthesis and Biological Evaluation of Halogenated AHL Analogues

#### 2.3.1. Synthesis of *N*-3-Oxoacylated Homoserine Lactones

**Scheme 2:**
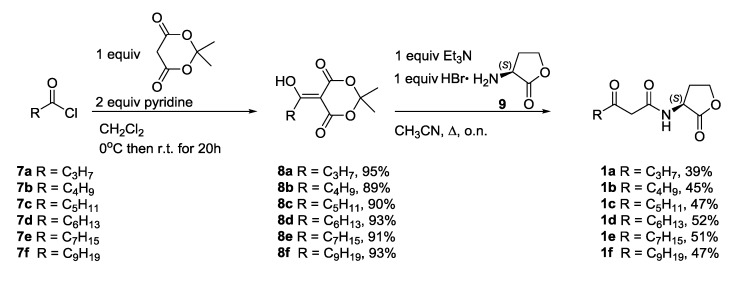
Synthesis of β-keto AHLs **1**.

(*S*)-Homoserine lactone hydrobromide **9** was prepared as previously described by reaction of l-methionine with bromoacetic acid [38]. β-Keto-AHLs **1a**–**f** were prepared by reaction of homoserine lactone hydrobromide **9** with adducts **8a**–**f**, which were initially prepared by reaction of Meldrum’s acid with the appropriate acid chloride **7a**–**f** (Scheme 2) [39]. This methodology allows the preparation of a wide variety of AHLs with a β-keto functionalized acyl chain in an efficient manner.

#### 2.3.2. Synthesis of Halogenated *N*-3-Oxoacylated Homoserine Lactone Analogues

**Scheme 3:**
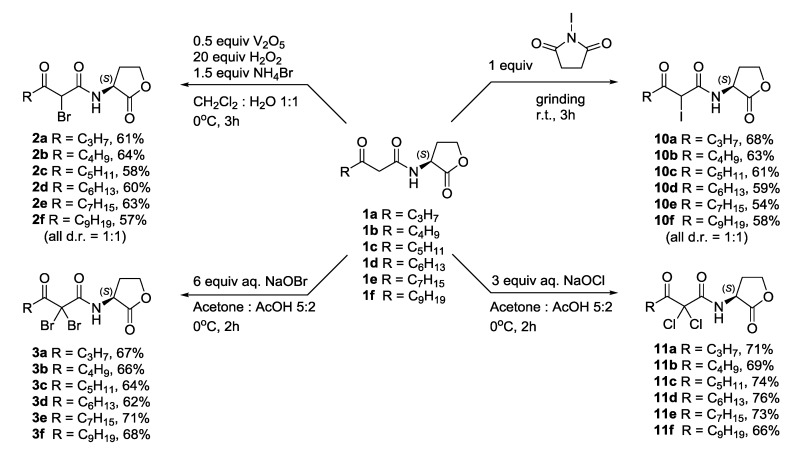
Synthesis of halogenated β-keto-AHL analogues.

In the next step, *N-*3-oxoacylated homoserine lactones **1a**–**f** were brominated chemoselectively to the corresponding α-monobrominated β-keto-AHLs **2a**–**f** by the use of a combination of vanadium pentoxide, ammonium bromide and hydrogen peroxide in a biphasic system of dichloromethane and water (Scheme 3) [40]. This transformation resembles the suggested natural process activated by vanadium-dependent haloperoxidases [5]. Alternatively, the α,α-dibrominated analogues **3a**–**f** were prepared by reaction of the corresponding β-keto-AHLs **1a**–**f** with a freshly prepared solution of sodium hypobromite [41]. Additionally, iodinated analogues **10a**–**f** were prepared by grinding the corresponding β-keto-AHL **1a**–**f** with *N-*iodosuccinimide (NIS) under neat conditions [42]. Finally, reaction of β-keto-AHLs **1a**–**f** with an aqueous solution of sodium hypochlorite resulted in the desired α,α-dichlorinated analogues **11a**–**f**.

**Scheme 4:**
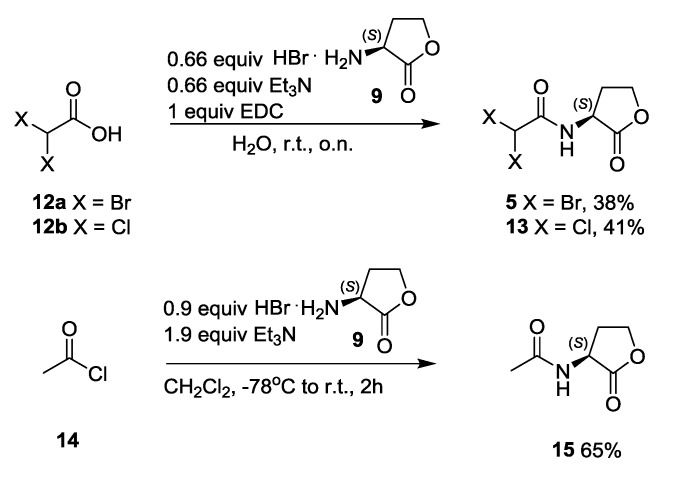
Synthesis of *N-*acetyl homoserine lactone **15** and halogenated derivatives **5** and **13**.

In a last step, the final cleavage products of the halogenated AHLs were prepared in acceptable yields by EDC-mediated coupling of the appropriate α,α-dihaloacetic acid **12a**,**b** with (*S*)-homoserine lactone hydrobromide **9** to yield *N*-α,α-dibromo- and *N*-α,α-dichloroacetyl homoserine lactones **5** and **13** in moderate yields (Scheme 4). Furthermore, *N*-acetyl homoserine lactone **15** was synthesized by reaction of acetyl chloride **14** with homoserine lactone hydrobromide **9** [38].

#### 2.3.3. Biological Evaluation

**Figure 2:**
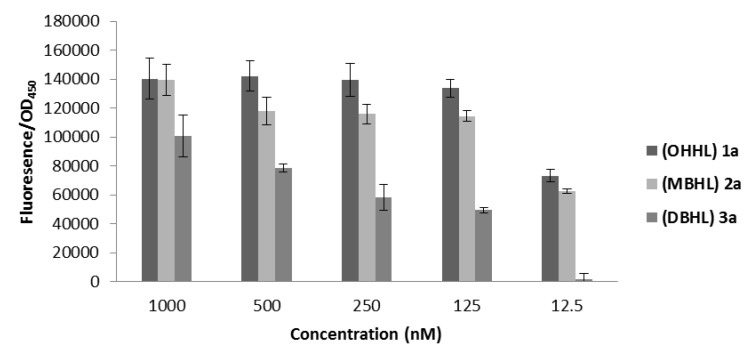
Quorum sensing-regulated green fluorescent protein (GFP) production by *Escherichia coli* JB523 induced by OHHL **1a** and the occuring degradation products. *N*-(α,α-Dibromoacetyl) homoserine lactone (DAHL) **5** was inactive in all concentrations. GFP production was determined by measuring the specific fluorescence. GFP fluorescence was corrected for the cell density of the reporter strain (fluorescence/OD_450_). Phosphate buffer saline was used as control. Results are expressed as mean value ± standard deviation of six repetitions.

In a next part of this research, all the synthesized compounds were tested for their ability to activate quorum sensing in the *E. coli* JB523 microplate assay. *E. coli* JB523 was selected as biosensor for this series of tests because of its relative high sensitivity and the ability to detect AHLs with various acyl chain lengths. The ability of the natural β-keto**-**AHLs **1**, their halogenated analogues **2**, **3**, **5**, **10**, **11**, **13** and *N*-acetyl homoserine lactone **15** to induce fluorescence was expressed as percentage of relative fluorescence (Supplementary Table S1). The QS stimulating activity of OHHL (**1a**) and the resulting degradation products **2a**, **3a** and **5** are demonstrated in Figure 2.

In most of the cases, the natural AHLs **1a**–**f** demonstrated agonistic activity in the entire concentration range (1000–1.25 nM). Not surprisingly, the activity of these natural AHLs **1** decreased with increasing length of the acyl chain (Supplementary Table S1). *N-*3-Oxohexanoyl and *N-*3-oxoheptanoyl homoserine lactone **1a** and **1b** showed the highest activity. Mono- and dibrominated analogues **2** and **3** were the most active compounds among the halogenated analogues. Frequently compounds **2** and **3** displayed activation in the whole concentration range. Nevertheless, their activity was reduced when compared to the natural AHL **1** with the same acyl chain length. Iodinated analogues **10a**–**f** demonstrated reduced activity when compared to their monobrominated analogues **2**, with the *N-*α-iodo-β-ketodecanoyl homoserine lactone **10e** being the most active among them. Surprisingly, the dichlorinated analogues **11a**–**f** with exception of **11d** and **11f** showed almost no activity. Moreover, the final degradation product **5**, the corresponding *N*-α,α-dichloroacetyl analogue **13** and *N**-***acetyl derivative **15**, were also evaluated as QS inducers. Overall, these compounds failed to induce GFP production. Since no activity was observed for *N*-acetyl homoserine lactone **15**, it can be suggested that cleavage of the acyl chain to an *N*-acetyl derivative is the crucial step for the complete loss of activity that was also observed in the *Chromobacterium violaceum* bioassay.

Subsequently, the halogenated analogues **2**, **3**, **5**, **10**, **11** and **13** were screened for their ability to disrupt QS in a competition test with the natural autoinducer OHHL **1a** in the *Escherichia coli* bioassay (Supplementary Table S2). However, none of the halogenated lactones was able to significantly reduce QS regulated GFP production in the whole concentration range (Supplementary Table S2). One might expect that due to the inductive effect of the halogen as an electrophilic moiety, a covalent bond could be formed between the halogenated analogue and the binding site of the receptor [43]. However, the absence of antagonistic activity of the novel halogenated analogues **2**, **3**, **10** and **11** indicates that the reduction in QS activity results from the presence of larger halogen substituents which causes changes in the steric and electronic properties of the *N*-acyl chain.

## 3. Experimental Section

### 3.1. Deactivation of *N*-Acylated Homoserine Lactones

#### 3.1.1. *N. cf pellucida* Culture

*N. cf pellucida* was isolated from the “Rammekenshoek” intertidal mudflat in the Westerschelde Estuary, The Netherlands (51°26′50′′N, 3°38′38′′E) on March 3, 2009. Cultures and permanent slides of the cultures used for the experiments were stored in DCG: BCCM 303 (Belgian Coordinated Collections of Microorganisms) Diatom Culture Collection hosted by the Laboratory of Protistology & Aquatic Ecology, Ghent University, Belgium. The culture medium was prepared by filtration and autoclavation of North Sea seawater which was supplemented with f/2 nutrients. The cultures were maintained in a climate room at 19 ± 1 °C and illuminated by cool-white fluorescent lamps at a rate of 50 μmol photons m^−2^s^−1^ with a light/dark cycle of 12/12 h.

#### 3.1.2. Phenol Red Assay

Haloperoxidase activity was verified via the bromination of phenol red (phenolsulfonphthalein) into bromophenol blue (3′,3′′,5′,5′′-tetrabromophenolsulfonphthalein). Phenol red (30 μM final concentration) was added to *N. cf pellucida* cultures 3h after daybreak. After two hours phenol red and bromophenol blue were monitored spectrophotometrically at 433 nm and 592 nm, respectively.

#### 3.1.3. *Chromobacterium violaceum* Bioassay

*Chromobacterium violaceum* CV026 is a mini-Tn5 mutant that is deficient in CviI (AHL synthase) which produces the purple pigment violacein in the presence of exogenous AHL [44]. This biosensor is most sensitive to HHL. A quantity of 10 mL of Luria-Bertani (LB) hard agar (1.5% agar) was placed in a Petri dish. The solidified agar was then covered with 5 mL of soft LB agar (0.5% agar) mixed with 100 µL of *Chromobacterium violaceum* CV026 culture (grown overnight at 27 °C). When the second layer was solidified, the agar was subsequently punched with a sterile glass pipette to form a well. Then 50 µL of test sample was removed from the appropriate culture and added to the well. Plates were incubated at 27 °C for 24 h and examined for violacein production. Violacein production was ranked visually from + + + (intense pigment, same as control with AHL added) to − (no pigment production, synthetic seawater was used as a negative control). For the deactivation experiment the conditions were: AHL (HHL or OHHL **1a**) concentration 5 ppm and H_2_O_2_ concentration 0.25 mM. For the catalase and BrCN experiments the conditions were the same as mentioned for the deactivation experiment.

#### 3.1.4. Catalase Experiment

The effect of the H_2_O_2_-decomposing enzyme catalase (600 units bovine liver catalase mL^−1^ dissolved in water, Sigma-Aldrich, St. Louis, MO, USA) on the QS deactivation by *N. cf pellucida* haloperoxidase was assessed by adding catalase together with H_2_O_2_ to *N. cf pellucida* cultures one hour before the onset of light and the *C. violaceum* bioassay was performed as described above, 3 h after the onset of light [33].

### 3.2. Degradation Pathway

#### 3.2.1. Liquid-Liquid Extraction of OHHL **1a** and Degradation Products

To active cultures of *N. cf pellucida*, 10 ppm of OHHL **1a** was added. The degradation of OHHL **1a** was followed over time (30 min; 60 min; 120 min; 180 min) by removing and filtering the culture medium with a GF/F filter. The culture medium was extracted twice with an equal volume of dichloromethane. The organic phases were collected and dried with MgSO_4_ and subsequently evaporated with a gentle stream of air. The dry extracts were stored in the freezer (−18 °C) prior to analysis.

#### 3.2.2. HPLC-MS Method

The analyses were performed with an Agilent 1100 LC-MSD system (Agilent Technologies, Waldbronn, Germany). The system was equipped with a quaternary pump, an autosampler, a vacuum degasser, and as a detector a Diode-array variable detector (DAD) and an 1100 6-port auto injector valve were used. The system was controlled by an Agilent software v.A.09.03. The Phenomenex C-18 (ODS, Octadecyl) security guard and the Phenomenex Luna C18 100A (Phenomenex, Torrance, CA, USA) column used were maintained at 35 °C. Elution was performed at a flow rate of 0.5 mL/min. As a mobile phase, a mixture of 0.02% acetic acid in water (solvent A) with pH = 3.1 and LC-MS grade acetonitrile (solvent B) were used. Detection of OHHL **1a** and the degradation products was performed at 220, 280 and 320 nm with a diode array detector. The mass spectrometer system was an Agilent 1100 equipped with an Agilent G1946D (SL) quadrapole mass spectrometer and electrospray ionization (ESI) system. As nebulizing gas, nitrogen was used at a pressure of 50 psi and with a flow of 13 L/min. The heated capillary was maintained at 350 °C and the voltage at 4.1 kV. The full mass spectra were measured from *m/z* 75 up to *m/z* 1000. The data were collected in positive ionization mode.

### 3.3. Chemical Synthesis

#### 3.3.1. Synthesis of Homoserine Lactone Hydrobromide **9**

A mixture of (*S*)-methionine (0.10 mol) and bromoacetic acid (0.11 mol) in 150 mL of a water-isopropanol-acetic acid mixture (5:5:2 v:v) was stirred at reflux overnight. The solvent was then removed under reduced pressure. Subsequently, the orange sticky oil was partly dissolved in 50 mL of a 4:1 mixture (v:v) of isopropanol:30% hydrogen bromide (HBr) in acetic acid. The desired compound was collected by filtration and the purification procedure was repeated starting by evaporation of the orange filtrate to dryness. Compound **9** was collected as a beige powder, mp 227–229 °C (mp 226–228 °C [38]). All other physical and analytical data were in agreement with literature data [38].

#### 3.3.2. Synthesis of β-Keto-AHLs **1a**–**f**

In a flame dried flask, Meldrum’s acid (36.9 mmol) and 6 mL of pyridine (74.2 mmol) were dissolved in dry dichloromethane (30 mL) under a nitrogen atmosphere. The appropriate acid chloride **7a**–**f** (36.9 mmol) was added dropwise at ice bath temperature. The mixture was stirred overnight, during which the temperature was allowed to increase to room temperature. Then, the reaction mixture was quenched with 1 M aq. HCl and the organic phase was washed with water and brine. The organic phase was collected and evaporated to yield adducts **8a**–**f**. The crude adducts **8a**–**f** were dissolved in acetonitrile, after which homoserine lactone hydrobromide **9** (36.9 mmol) and Et_3_N (36.9 mmol) were added and the reaction mixture was stirred overnight at reflux. The organic phase was evaporated and the crude product was dissolved in ethyl acetate. The organic phase was washed with 1M aq. HCl and brine. The organic phase was dried with MgSO_4_, evaporated and the crude product was purified via column chromatography on silica gel (EtOAc:petroleum ether 3:2) to yield the desired β-keto-AHLs **1a**–**f**.

#### 3.3.3. Synthesis of α-Bromo-β-keto-AHLs **2a**–**f**

To a stirred solution of vanadium pentoxide (1.9 mmol) in water was added 50% solution of hydrogen peroxide in water (76 mmol) at ice-bath temperature while stirring. The color changed from light orange to deep red after 25–30 min. Then, ammonium bromide (5.7 mmol) was added and the reaction mixture was stirred for another 10 min. Subsequently*,* β-keto-AHL **1a**–**f** (3.8 mmol) was added in dichloromethane and the reaction mixture was then stirred further for 3 h at the same temperature. After completion of the reaction, as monitored by TLC, the mixture was extracted twice with dichloromethane (25 mL) and the organic layer was washed with saturated sodium metabisulfite solution (15 mL). Finally, it was washed with water and dried with MgSO_4_. The organic phase was evaporated and the crude products were purified via column chromatography on silica gel (EtOAc:petroleum ether 4:1) to yield the desired **2a**–**f**.

#### 3.3.4. Synthesis of α,α-Dichloro-β-keto and α,α-Dibromo-β-keto-AHLs **11a**–**f** and **3a**–**f**

To a solution of the appropriate β-keto*-*AHL **1a**–**f** (1.20 mmol) in acetone (5 mL) and glacial acetic acid (2 mL) cooled to 0 °C was added dropwise sodium hypochlorite solution (3.6 mmol). The mixture was stirred for 2 h at 0 °C, then poured into saturated Na_2_CO_3_ solution and extracted with dichloromethane. The combined organic layers were dried (MgSO_4_) and concentrated *in vacuo*. The crude product was purified by flash chromatography on silica gel (EtOAc:petroleum ether 2:3) to afford the dichlorinated products **11a**–**f**. Brominations were conducted as described above using freshly prepared sodium hypobromite solution (7.2 mmol). The stock solution of sodium hypobromite was prepared by slowly adding bromine (16.6 mmol) to a solution of sodium hydroxide (50 mmol) in water (25 mL) at 0 °C. The mixture was stirred for 15 min and used immediately.

#### 3.3.5. Synthesis of α-Iodo-β-keto-AHLs **10a**–**f**

In a porcelain mortar 1 mmol of the appropriate β-keto-AHL **1a**–**f** was ground for 5 min with *N-*iodosuccinimide (1 mmol) and the mixture was left to react at room temperature for 3 h. The formed paste was subsequently extracted with dichloromethane and the organic phase was washed with water. The collected organic phase was dried with MgSO_4_ and evaporated. Crude products were purified by column chromatography on silica gel (EtOAc:petroleum ether 4:1) to yield the desired α-iodo-β-keto AHLs **10***.*

#### 3.3.6. Synthesis of *N*-α,α-Dibromo- and *N*-α,α-Dichloroacetyl Homoserine Lactones **5** and **13**

To a stirred solution of (*S*)-homoserine lactone hydrobromide **9** (2 mmol) in 5 mL of water, triethylamine (2 mmol) was added, followed by the addition of the appropriate brominated or chlorinated acetic acid **12** (3 mmol) and 1-ethyl-3-(3-dimethylaminopropyl)carbodiimide hydrochloride (3 mmol). The mixture was stirred overnight at room temperature. The aqueous phase was extracted two times with ethyl acetate (20 mL) and the organic phase was washed with saturated aq. NaHCO_3_ solution and brine. Drying with MgSO_4_, filtration and evaporation of the solvent gave the corresponding halogenated acetyl homoserine lactones **5** and **13**. The crude product was purified via column chromatography on silica gel (EtOAc:petroleum ether 4:1).

#### 3.3.7. Synthesis of *N*-Acetyl Homoserine Lactone **15**

Under dry conditions, triethylamine (3.8 mmol) was added to a stirred solution of (*S*)-homoserine lactone hydrobromide **9** (1.8 mmol) in 50 mL dichloromethane cooled at −78 °C. Acetyl chloride **14** (2 mmol) was then added dropwise and the temperature was allowed to rise to room temperature during a period of 2 h. Evaporation to dryness gave a white semi-solid which was partly dissolved by ethyl acetate and the salt remaining was separated by filtration. The organic phase was evaporated and purified by column chromatography on silica gel (EtOAc:petroleum ether 4:1).

### 3.4. Biological Activity of Novel Halogenated AHL Analogues

#### *Escherichia coli* JB523 Green Fluorescent Protein (GFP) Microplate Assay

*E. coli* JB523 is a highly sensitive biosensor which contains plasmid pJBA130 that encodes green fluorescent protein (GFP) in response to exogenous AHL [45]. Strain JB523 was grown overnight at 28 °C in LB medium supplemented with 20 mg/L tetracycline until the optical density (OD) reached approximately 1 at 550 nm. The bacteria were then diluted to an OD_600_ of 0.1. Then 100 μL of the diluted culture was mixed with 100 μL of the appropriate concentration of the tested compound. Phosphate buffer saline (PBS) was used to prepare the stock solutions for all concentrations. PBS was used as a negative control for activation tests. For the inhibition tests, the procedure was the same with the exception of the addition of 100 μL of a 100 nM solution OHHL **1a** to the culture medium to reach a final concentration of 50 nM. The plate was gently stirred in an incubator at 28 °C for 2 h. QS-regulated GFP production was then assessed by fluorescence measurements (excitation at 475 nm and emission at 515 nm) using a PerkinElmer VICTOR X (PerkinElmer, Waltham, MA, USA) multilabel plate reader. The fluorescence was normalized for cell density of the reporter strain.

## 4. Conclusions

In conclusion, the deactivation of β-keto-AHLs by the natural haloperoxidase system of the benthic diatom *N. cf pellucida* has been reported for the first time. The disruption of these signal molecules was shown to be H_2_O_2_-dependent. In addition, the degradation pathway towards halogenated derivatives was elucidated by means of HPLC-MS and by the synthesis of a broad library of novel halogenated AHL analogues as reference compounds. The ability of these AHL analogues as quorum sensing modulators was directly compared to and evaluated against a series of naturally occurring β-keto AHLs. Finally, it was demonstrated that the haloperoxidase mediated loss of QS activity resulted from the final cleavage of the halogenated *N*-acyl chain of the signal molecules.

## Supplementary Files

Supplementary File 1 Supplementary Information (PDF, 387 KB)
