# Supplementary material for: Haloperoxidase Mediated Quorum Quenching by Nitzschia cf pellucida: Study of the Metabolization of N-Acyl Homoserine Lactones by a Benthic Diatom
Source: Mar Drugs. 2014 Jan 17;12(1):352–67. doi: 10.3390/md12010352 (PMC3917277; doi:10.3390/md12010352)
Supplement: Supplementary File 1 — Supplementary Information (PDF, 387 KB) [file marinedrugs-12-00352-s001.pdf]

Supplementary Information

Table S1. Quorum sensing-regulated GFP production by *Escherichia coli* JB523 induced by natural AHLs **1** and haloacylated analogues **2**, **3**, **5**, **10**,**11**, **13** and **15**.

	1000 nM		500 nM		250 nM		125 nM		12.5 nM		1.25 nM	
	**Mean**	**St Dev**	**Mean**	**St Dev**	**Mean**	**St Dev**	**Mean**	**St Dev**	**Mean**	**St Dev**	**Mean**	**St Dev**
**1a**	191.7	2.9	194.0	2.4	190.2	2.1	182.8	5.4	100.0	6.3	55.0	3.5
**2a**	190.6	7.9	161.2	7.4	158.4	7.6	156.4	18.0	85.4	22.0	38.0	2.6
**3a**	137.8	30.6	107.6	18.3	79.3	6.1	67.4	4.3	2.5	2.8	NA	–
**10a**	38.5	23.1	19.5	11.7	14.2	7.2	14.3	3.5	13.4	0.2	NA	–
**11a**	NA	–	NA	–	NA	–	NA	–	NA	–	NA	–
**1b**	69.3	8.3	67.5	2.6	65.0	3.4	57.5	3.0	43.7	3.4	41.0	6.8
**2b**	7.5	34.9	6.5	22.0	8.8	31.2	8.4	35.0	9.5	18.2	NA	–
**3b**	52.9	9.7	49.5	2.5	39.8	50.6	32.4	4.1	13.2	22.9	1.2	2.8
**10b**	56.0	4.8	59.9	3.0	51.8	4.3	29.9	4.8	1.3	6.9	NA	–
**11b**	NA	–	9.3	3.2	5.7	1.4	9.8	0.3	10.3	0.4	NA	–
**1c**	42.3	1.4	45.1	1.4	37.7	1.8	30.2	0.6	10.3	0.7	2.9	1.7
**2c**	31.9	12.9	31.6	6.8	17.5	3.7	26.0	5.5	NA	–	NA	–
**3c**	6.1	1.8	11.1	1.3	7.0	1.7	23.5	1.3	NA	–	NA	–
**10c**	NA	–	4.0	1.7	NA	4.2	1.2	2.6	NA	–	NA	–
**11c**	NA	–	NA	–	NA	–	NA	–	NA	–	NA	–
**1d**	64.5	4.7	53.4	7.1	57.6	3.6	61.0	4.3	45.9	5.2	28.7	7.0
**2d**	19.7	4.0	15.6	2.6	16.7	5.1	15.1	4.4	8.6	4.9	NA	–
**3d**	NA	–	NA	–	18.9	5.9	13.7	6.5	2.7	6.8	NA	–
**10d**	29.5	3.0	28.2	4.2	17.6	7.8	9.4	6.8	NA	–	NA	–
**11d**	41.8	3.3	36.8	2.7	40.0	2.2	41.6	3.2	21.1	3.4	8.3	7.8
**1e**	81.0	5.5	52.4	1.4	54.7	2.8	54.9	3.0	29.3	14.2	NA	–
**2e**	15.6	2.3	NA	–	NA	–	2.0	3.5	NA	–	NA	–
**3e**	22.2	14.5	3.1	13.6	NA	–	NA	–	NA	–	NA	–
**10e**	57.5	2.6	40.2	1.1	26.8	1.3	20.6	1.2	11.4	5.9	5.2	3.4
**11e**	47.1	2.8	40.4	3.8	41.3	4.2	38.0	1.6	25.2	2.3	10.8	6.3

Table S1. *Cont.*

**1f**	58.3	51.0	44.4	9.6	21.7	18.8	15.7	22.3	2.1	6.5	NA	–
**2f**	28.7	2.4	25.9	3.2	29.8	4.7	31.9	5.5	26.8	2.3	13.4	12.0
**3f**	35.8	5.3	33.4	2.2	33.3	1.8	37.9	5.3	26.1	6.2	9.6	18.0
**10f**	3.5	48.0	0.6	3.9	NA	–	0.5	5.3	NA	–	NA	–
**11f**	NA	–	NA	–	NA	–	NA	–	NA	–	NA	–
**5**	NA	–	NA	–	NA	–	NA	–	NA	–	NA	–
**13**	NA	–	NA	–	NA	–	NA	–	NA	–	NA	–
**15**	NA	–	NA	–	NA	–	NA	–	NA	–	NA	–

GFP production was determined by measuring specific fluorescence. GFP fluorescence was corrected for cell density of the reporter strain (Fluorescence/OD 550 nm). Phosphate buffer saline was used as control; the specific fluorescence observed for 12.5 nM of OHHL was set at 100% and the other values were normalized accordingly. Results are expressed as mean value ± standard deviation of six repetitions. The intensity of the color in the table is related to the relative amount of GFP production. NA: not active.

Table S2. Inhibition of quorum sensing-regulated GFP production by *Escherichia coli* JB523 by haloacylated analogues **2**, **3**, **5**, **10**, **11** and **13**.

	**1000 nM**		**750 nM**		**500 nM**		**250 nM**		**100 nM**		**10 nM**		**1 nM**	
	**Mean**	**St Dev**	**Mean**	**St Dev**	**Mean**	**St Dev**	**Mean**	**St Dev**	**Mean**	**St Dev**	**Mean**	**St Dev**	**Mean**	**St Dev**
**2a**	105.4	4.1	104.5	2.7	97.7	3.0	99.5	3.2	99.6	4.7	93.5	9.3	79.7	14.5
**3a**	101.5	2.7	97.9	2.8	97.2	5.0	97.8	2.2	94.8	2.3	96.2	1.8	106.1	4.6
**10a**	105.3	9.0	109.4	21.5	100.8	2.0	100.6	2.4	101.0	3.1	101.7	3.4	107.5	2.0
**11a**	102.6	3.7	102.1	2.1	101.6	1.8	102.4	1.9	102.4	1.5	105.3	1.2	103.8	1.7
**2b**	96.5	5.8	98.3	1.9	98.6	2.9	100.4	3.8	100.4	3.3	99.2	3.4	95.5	2.3
**3b**	99.5	4.0	98.2	3.1	99.0	3.2	97.5	4.9	94.6	3.0	94.9	2.2	104.2	3.5
**10b**	103.8	5.7	116.7	29.6	101.1	2.0	100.9	2.0	100.3	2.7	93.6	2.6	97.2	4.5
**11b**	92.3	7.1	93.9	3.0	93.4	2.7	96.3	4.3	93.2	7.0	96.7	1.8	91.2	3.0
**2c**	104.0	6.9	95.7	2.3	98.1	1.9	96.7	3.0	96.4	2.1	94.8	2.6	106.3	1.1
**3c**	96.0	2.5	96.7	1.7	96.7	3.9	94.0	3.0	94.4	2.7	93.9	2.6	115.7	5.3
**10c**	103.9	2.0	99.0	2.8	98.5	2.4	99.3	0.8	97.4	1.7	96.3	1.6	96.7	0.5

Table S2. *Cont.*

**11c**	97.3	2.0	98.0	1.1	97.6	1.5	97.0	1.5	97.6	0.8	99.4	1.4	109.1	4.0
**2d**	99.2	2.7	94.4	0.9	94.1	1.7	93.8	1.5	92.4	1.0	93.0	3.4	110.6	1.8
**3d**	100.6	15.4	95.6	2.9	94.9	2.3	93.4	3.8	96.0	3.1	100.3	5.8	118.2	9.8
**10d**	102.5	2.4	101.7	0.6	98.0	7.2	104.7	9.2	95.8	3.6	94.1	3.9	99.9	2.5
**11d**	96.5	4.9	96.9	5.4	95.9	6.5	95.6	7.0	96.7	6.8	97.4	7.0	111.3	2.0
**2e**	101.6	8.0	97.1	2.5	95.2	3.2	94.7	2.3	94.7	1.5	94.2	3.2	112.4	1.3
**3e**	95.9	3.1	95.9	2.7	98.2	6.8	97.8	1.5	100.5	1.8	117.7	8.8	112.9	6.1
**10e**	112.4	8.7	104.0	1.5	103.9	0.7	102.0	3.1	103.1	1.6	100.2	2.2	110.7	1.0
**11e**	101.9	2.4	102.3	1.8	100.2	1.8	102.0	1.2	102.2	1.4	103.2	1.7	101.1	3.2
**2f**	101.4	5.7	96.8	3.0	98.5	7.2	96.6	2.5	96.9	3.2	93.5	3.6	107.4	7.2
**3f**	98.4	1.6	98.8	4.7	97.4	4.2	95.6	1.9	96.9	3.1	98.6	1.3	105.6	1.9
**10f**	96.3	2.8	96.4	2.2	95.7	4.0	96.4	2.3	96.3	1.7	97.9	1.4	110.9	1.3
**11f**	97.5	3.0	95.6	2.2	93.0	7.7	95.9	2.3	92.9	10.4	96.3	2.2	103.6	0.8
**5**	97.3	2.8	97.4	2.5	96.3	4.1	95.2	2.9	95.9	2.9	97.5	3.0	96.8	7.3
**13**	100.2	3.4	97.4	11.2	98.5	1.7	94.8	3.9	94.2	8.0	102.0	9.6	110.6	7.4

Inhibition of quorum sensing-regulated GFP production by *Escherichia coli* JB523 by α-haloacylated analogues **2**, **3**, **5**, **10**, **11** and **13** in the presence of 50 nM of OHHL. GFP production was determined by measuring specific fluorescence. GFP fluorescence was corrected for cell density of the reporter strain (Fluorescence/OD 550 nm). Phosphate buffer saline was used as control; the specific fluorescence observed for 50 nM of OHHL was set at 100% and the other values were normalized accordingly. Results are expressed as mean value ± standard deviation of six repetitions. The intensity of the color in the table is related to the relative amount of GFP production.

S1. Characterization of Novel Compounds

S1.1. N*-(2-Bromo-3-oxohexanoyl)-(*S*)-homoserine Lactone* **2a**

S1.1.1. (IUPAC: 2-Bromo-3-oxo-*N*-[(3*S*)-tetrahydro-2-oxo-3-furanyl]-hexanamide)

^{1}H NMR (300 MHz, $CDCl_3$): δ 0.94 (3H, t, *J* = 7.2 Hz, $C\underline{H}_{3\text{ isomer 1 and 2}}$); 1.67 (2H, sext, *J* = 7.2 Hz, $C\underline{H}_2CH_{3\text{ isomer 1 and 2}}$); 2.27 (1H, dddd, *J* = 11.8 Hz, 11.7 Hz, 11.7 Hz, 9.1 Hz, $OCH_2C\underline{H}H'_{\text{isomer 1 and 2}}$); 2.72 (2H, t, *J* = 7.2 Hz, $C\underline{H}_2C{=}O_{\text{isomer 1 and 2}}$); 2.73–2.79 (1H, m, $OCH_2CH\underline{H}'_{\text{isomer 1 and 2}}$); 4.30 (1H, ddd, *J* = 11.0 Hz, 9.4 Hz, 6.1 Hz, $OC\underline{H}H'_{\text{isomer 1 and 2}}$); 4.50 (1H, t, *J* = 9.1 Hz, $OCH\underline{H}'_{\text{isomer 1 and 2}}$); 4.51–4.58 (1H, m, $C\underline{H}N_{\text{isomer 1 and 2}}$); 4.76 (0.5H, s, $C\underline{H}Br_{\text{isomer 1}}$); 4.79 (0.5H, s, $C\underline{H}Br_{\text{isomer 2}}$); 7.39 (1H, d, *J* = 6.1 Hz, $N\underline{H}_{\text{isomer 1 and 2}}$).

^{13}C NMR (75 MHz, $CDCl_3$): δ 13.3 ($\underline{C}H_{3\text{ isomer 1 and 2}}$); 17.0 ($\underline{C}H_2CH_{3\text{ isomer 1 and 2}}$); 29.60 ($\underline{C}H_2CHN_{\text{isomer 1}}$); 29.65 ($\underline{C}H_2CHN_{\text{isomer 2}}$); 42.0 ($\underline{C}H_2C{=}O_{\text{isomer 1}}$); 42.1 ($\underline{C}H_2C{=}O_{\text{isomer 2}}$); 48.0 ($\underline{C}HBr_{\text{isomer 1}}$); 48.1 ($\underline{C}HBr_{\text{isomer 2}}$); 49.80 ($\underline{C}HN_{\text{isomer 1}}$); 49.90 ($\underline{C}HN_{\text{isomer 2}}$); 65.8 ($\underline{C}H_2O_{\text{isomer 1 and 2}}$); 164.8 ($N\underline{C}{=}O_{\text{isomer 1}}$); 165.0 ($N\underline{C}{=}O_{\text{isomer 2}}$); 174.1 ($O\underline{C}{=}O_{\text{isomer 1}}$); 174.2 ($O\underline{C}{=}O_{\text{isomer 2}}$); 200.3 ($CH_2\underline{C}{=}O_{\text{isomer 1}}$); 200.5 ($CH_2\underline{C}{=}O_{\text{isomer 2}}$). **MS (ESI):** *m/z* (%): 292/294 ($M + H^+$, 30).

HRMS mass calculated: $C_{10}H_{14}BrNO_4H^+$: 292.0184; **obtained:** 292.0177. **IR (cm^{-1}) ν_{max}:** 1018; 1174 (C-O); 1546 (HN-C=O); 1652 (HN-C=O); 1730 ($C{=}O_{\text{ketone}}$); 1774 ($C{=}O_{\text{lactone}}$); 2878 (CH); 2964 (CH); 3070 (CH); 3287 (NH). **Melting Point:** 148 °C. White powder. **Y:** 61%.

S1.2. N*-(2-Bromo-3-oxoheptanoyl)-(*S*)-homoserine Lactone* **2b**

S1.2.1. (IUPAC: 2-Bromo-3-oxo-*N*-[(3*S*)-tetrahydro-2-oxo-3-furanyl]-heptanamide)

^{1}H NMR (300 MHz, $CDCl_3$): δ 0.92 (3H, t, *J* = 7.2Hz, $C\underline{H}_{3\text{ isomer 1 and 2}}$); 1.34 (2H, sext, *J* = 7.2 Hz, $C\underline{H}_2CH_{3\text{ isomer 1 and 2}}$); 1.62 (2H, quint, *J* = 7.2 Hz, $C\underline{H}_2CH_2CH_{3\text{ isomer 1 and 2}}$); 2.28 (1H, dddd, *J* = 11.8 Hz, 11.8 Hz, 11.8 Hz, 9.2 Hz, $OCH_2C\underline{H}H'_{\text{isomer 1 and 2}}$); 2.76 (2H, t, *J* = 7.2 Hz, $C\underline{H}_2C{=}O_{\text{isomer 1 and 2}}$); 2.79-2.87 (1H, m, $OCH_2CH\underline{H}'_{\text{isomer 1 and 2}}$); 4.31 (1H, ddd, *J* = 11.0 Hz, 9.4 Hz, 6.1 Hz, $OC\underline{H}H'_{\text{isomer 1 and 2}}$); 4.51 (1H, t, *J* = 9.2 Hz, $OCH\underline{H}'_{\text{isomer 1 and 2}}$); 4.52-4.61 (1H, m, $C\underline{H}N_{\text{isomer 1 and 2}}$); 4.80 (0.5H, s, $C\underline{H}Br_{\text{isomer 1}}$); 4.83 (0.5H, s, $C\underline{H}Br_{\text{isomer 2}}$); 7.27 (0.5H, d, *J* = 6.1 Hz, $N\underline{H}_{\text{isomer 1}}$); 7.34 (0.5H, d, *J* = 6.1 Hz, $N\underline{H}_{\text{isomer 2}}$).

^{13}C NMR (75 MHz, $CDCl_3$): δ 13.8 ($\underline{C}H_{3\text{ isomer 1 and 2}}$); 22.0 ($\underline{C}H_2CH_{3\text{ isomer 1 and 2}}$); 25.7 ($\underline{C}H_2CH_2CH_{3\text{ isomer 1 and 2}}$); 29.54 ($\underline{C}H_2CHN_{\text{isomer 1}}$); 29.57 ($\underline{C}H_2CHN_{\text{isomer 2}}$); 39.9 ($\underline{C}H_2C{=}O_{\text{isomer 1}}$); 40.1 ($\underline{C}H_2C{=}O_{\text{isomer 2}}$); 47.9 ($\underline{C}HBr_{\text{isomer 1}}$); 48.3 ($\underline{C}HBr_{\text{isomer 2}}$); 49.88 ($\underline{C}HN_{\text{isomer 1}}$); 49.94 ($\underline{C}HN_{\text{isomer 2}}$); 66.1 ($\underline{C}H_2O_{\text{isomer 1 and 2}}$); 164.9 ($N\underline{C}{=}O_{\text{isomer 1}}$); 165.0 ($N\underline{C}{=}O_{\text{isomer 2}}$); 174.2 ($O\underline{C}{=}O_{\text{isomer 1}}$); 174.3 ($O\underline{C}{=}O_{\text{isomer 2}}$); 200.3 ($CH_2\underline{C}{=}O_{\text{isomer 1}}$); 200.6 ($CH_2\underline{C}{=}O_{\text{isomer 2}}$). **MS (ESI):** *m/z* (%): 306/308 ($M + H^+$, 16); 323/325($M + NH_4^+$, 100).

HRMS mass calculated: $C_{11}H_{16}BrNO_4H^+$: 306.03410; **obtained:** 306.0326. **IR (cm^{-1}) ν_{max}:** 1017; 1173 (C-O); 1546 (HN-C=O); 1651 (HN-C=O); 1730 ($C{=}O_{\text{ketone}}$); 1773 ($C{=}O_{\text{lactone}}$); 2872 (CH); 2934 (CH); 2957 (CH); 3283 (NH). **Melting Point:** 152 °C. White powder. **Y:** 64%.

S1.3. N*-(2-Bromo-3-oxooctanoyl)-(*S*)-homoserine Lactone* **2c**

S1.3.1. (IUPAC: 2-Bromo-3-oxo-*N*-[(3*S*)-tetrahydro-2-oxo-3-furanyl]-octanamide)

^{1}H NMR (300 MHz, $CDCl_3$): δ 0.89 (3H, t, J = 7.2Hz, $C\underline{H}_{3\text{ isomer 1 and 2}}$); 1.24–1.36 (4H, m, $(C\underline{H}_2)_2CH_{3\text{ isomer 1 and 2}}$); 1.63 (2H, quint, J = 7.2 Hz, $C\underline{H}_2(CH_2)_2CH_{3\text{ isomer 1 and 2}}$); 2.27 (1H, dddd, J = 11.8 Hz, 11.7 Hz, 11.7 Hz, 9.1 Hz, $OCH_2C\underline{H}H'_{\text{isomer 1 and 2}}$); 2.75 (2H, t, J = 7.2 Hz, $C\underline{H}_2C{=}O_{\text{isomer 1 and 2}}$); 2.79–2.86 (1H, m, $OCH_2CH\underline{H}'_{\text{isomer 1 and 2}}$); 4.31 (1H, ddd, J = 11.0 Hz, 9.4 Hz, 6.1 Hz, $OC\underline{H}H'_{\text{isomer 1 and 2}}$); 4.51 (1H, t, J = 9.0 Hz, $OCH\underline{H}'_{\text{isomer 1 and 2}}$); 4.52-4.60 (1H, m, $C\underline{H}N_{\text{isomer 1 and 2}}$); 4.78 (0.5H, s, $C\underline{H}Br_{\text{isomer 1}}$); 4.81 (0.5H, s, $C\underline{H}Br_{\text{isomer 2}}$); 7.39 (1H, d, J = 6.1 Hz, $N\underline{H}_{\text{isomer 1 and 2}}$).

^{13}C NMR (75 MHz, $CDCl_3$): δ 13.9 ($\underline{C}H_{3\text{ isomer 1 and 2}}$); 23.2; 23.7 and 31.0 ($(\underline{C}H_2)_3CH_{3\text{ isomer 1 and 2}}$); 29.50 ($\underline{C}H_2CHN_{\text{isomer 1}}$); 29.55 ($\underline{C}H_2CHN_{\text{isomer 2}}$); 40.1 ($\underline{C}H_2C{=}O_{\text{isomer 1}}$); 40.3 ($\underline{C}H_2C{=}O_{\text{isomer 2}}$); 47.7 ($\underline{C}HBr_{\text{isomer 1}}$); 48.1 ($\underline{C}HBr_{\text{isomer 2}}$); 49.80 ($\underline{C}HN_{\text{isomer 1}}$); 49.86 ($\underline{C}HN_{\text{isomer 2}}$); 66.0 ($\underline{C}H_2O_{\text{isomer 1 and 2}}$); 164.8 ($N\underline{C}{=}O_{\text{isomer 1}}$); 165.0 ($N\underline{C}{=}O_{\text{isomer 2}}$); 174.1 ($O\underline{C}{=}O_{\text{isomer 1}}$); 174.2 ($O\underline{C}{=}O_{\text{isomer 2}}$); 200.3 ($CH_2\underline{C}{=}O_{\text{isomer 1}}$); 200.5 ($CH_2\underline{C}{=}O_{\text{isomer 2}}$). **MS (ESI):** *m/z* (%): 320/322 ($M + H^+$, 30).

HRMS mass calculated: $C_{12}H_{18}BrNO_4H^+$: 320.0497; **obtained:** 320.0476. **IR (cm^{-1}) ν_{max}:** 1017; 1174 (C-O); 1546 (HN-C=O); 1652 (HN-C=O); 1731 ($C{=}O_{ketone}$); 1774 ($C{=}O_{lactone}$); 2874 (CH); 2961 (CH); 3065 (CH); 3287 (NH). **Melting Point:** 150 °C. White powder. **Y:** 58%.

S1.4. N*-(2-Bromo-3-oxononanoyl)-(*S*)-homoserine Lactone* **2d**

S1.4.1. (IUPAC: 2-Bromo-3-oxo-*N*-[(3*S*)-tetrahydro-2-oxo-3-furanyl]-nonanamide)

^{1}H NMR (300 MHz, $CDCl_3$): δ 0.88 (3H, t, J = 7.2Hz, $C\underline{H}_{3\text{ isomer 1 and 2}}$); 1.21–1.41 (6H, m, $(C\underline{H}_2)_3CH_{3\text{ isomer 1 and 2}}$); 1.63 (2H, quint, J = 7.2 Hz, $C\underline{H}_2(CH_2)_3CH_{3\text{ isomer 1 and 2}}$); 2.27 (1H, dddd, J = 11.8 Hz, 11.7 Hz, 11.7 Hz, 9.1 Hz, $OCH_2C\underline{H}H'_{\text{isomer 1 and 2}}$); 2.75 (2H, t, J = 7.2 Hz, $C\underline{H}_2C{=}O_{\text{isomer 1 and 2}}$); 2.79–2.92 (1H, m, $OCH_2CH\underline{H}'_{\text{isomer 1 and 2}}$); 4.31 (1H, ddd, J = 11.0 Hz, 9.4 Hz, 6.1 Hz, $OC\underline{H}H'_{\text{isomer 1 and 2}}$); 4.51 (1H, t, J = 9.0 Hz, $OCH\underline{H}'_{\text{isomer 1 and 2}}$); 4.52-4.60 (1H, m, $C\underline{H}N_{\text{isomer 1 and 2}}$); 4.79 (0.5H, s, $C\underline{H}Br_{\text{isomer 1}}$); 4.82 (0.5H, s, $C\underline{H}Br_{\text{isomer 2}}$); 7.27 (0.5H, d, J = 6.1 Hz, $N\underline{H}_{\text{isomer 1}}$); 7.34 (0.5H, d, J = 6.1 Hz, $N\underline{H}_{\text{isomer 2}}$).

^{13}C NMR (75 MHz, $CDCl_3$): δ 14.1 ($\underline{C}H_{3\text{ isomer 1 and 2}}$); 22.5; 23.7; 28.6 and 31.5 ($(\underline{C}H_2)_4CH_{3\text{ isomer 1 and 2}}$); 29.60 ($\underline{C}H_2CHN_{\text{isomer 1}}$); 29.65 ($\underline{C}H_2CHN_{\text{isomer 2}}$); 40.3 ($\underline{C}H_2C{=}O_{\text{isomer 1}}$); 40.4 ($\underline{C}H_2C{=}O_{\text{isomer 2}}$); 47.8 ($\underline{C}HBr_{\text{isomer 1}}$); 48.2 ($\underline{C}HBr_{\text{isomer 2}}$); 49.88 ($\underline{C}HN_{\text{isomer 1}}$); 49.94 ($\underline{C}HN_{\text{isomer 2}}$); 66.1 ($\underline{C}H_2O_{\text{isomer 1 and 2}}$); 164.9 ($N\underline{C}{=}O_{\text{isomer 1}}$); 165.1 ($N\underline{C}{=}O_{\text{isomer 2}}$); 174.2 ($O\underline{C}{=}O_{\text{isomer 1}}$); 174.3 ($O\underline{C}{=}O_{\text{isomer 2}}$); 200.3 ($CH_2\underline{C}{=}O_{\text{isomer 1}}$); 200.5 ($CH_2\underline{C}{=}O_{\text{isomer 2}}$). **MS (ESI):** *m/z* (%): 334/336 ($M + H^+$, 30); 351/353 ($M + NH_4^+$, 100).

HRMS mass calculated: $C_{13}H_{20}BrNO_4H^+$: 334.0654; **obtained:** 334.0647. **IR (cm^{-1}) ν_{max}:** 1019; 1174 (C-O); 1545 (HN-C=O); 1651 (HN-C=O); 1730 ($C{=}O_{ketone}$); 1774 ($C{=}O_{lactone}$); 2853 (CH); 2928 (CH); 2961 (CH); 3286 (NH). **Melting Point:** 155 °C. White powder. **Y:** 60%.

S1.5. N*-(2-Bromo-3-oxodecanoyl)-(*S*)-homoserine Lactone* **2e**

S1.5.1. (IUPAC: 2-Bromo-3-oxo-*N*-[(3*S*)-tetrahydro-2-oxo-3-furanyl]-decanamide)

^{1}H NMR (300 MHz, $CDCl_3$): δ 0.88 (3H, t, *J* = 7.2Hz, $C\underline{H}_{3\text{ isomer 1 and 2}}$); 1.24–1.33 (8H, m, $(C\underline{H}_2)_4CH_{3\text{ isomer 1 and 2}}$); 1.58-1.66 (2H, m, $C\underline{H}_2(CH_2)_4CH_{3\text{ isomer 1 and 2}}$); 2.25 (1H, dddd, *J* = 11.1 Hz, 11.1 Hz, 11.1 Hz, 9.1 Hz, $OCH_2C\underline{H}H'_{\text{isomer 1 and 2}}$); 2.75 (2H, t, *J* = 7.2 Hz, $C\underline{H}_2C{=}O_{\text{isomer 1 and 2}}$); 2.79–2.89 (1H, m, $OCH_2CH\underline{H}'_{\text{isomer 1 and 2}}$); 4.31 (1H, ddd, *J* = 11.0 Hz, 9.4 Hz, 6.1 Hz, $OC\underline{H}H'_{\text{isomer 1 and 2}}$); 4.51 (1H, t, *J* = 9.0 Hz, $OCH\underline{H}'_{\text{isomer 1 and 2}}$); 4.52–4.60 (1H, m, $C\underline{H}N_{\text{isomer 1 and 2}}$); 4.77 (0.5H, s, $C\underline{H}Br_{\text{isomer 1}}$); 4.80 (0.5H, s, $C\underline{H}Br_{\text{isomer 2}}$); 7.20 (0.5H, d, *J* = 6.1 Hz, $N\underline{H}_{\text{isomer 1}}$); 7.21 (0.5H, d, *J* = 6.1 Hz, $N\underline{H}_{\text{isomer 2}}$).

^{13}C NMR (75 MHz, $CDCl_3$): δ 14.4 ($\underline{C}H_{3\text{ isomer 1 and 2}}$); 22.6; 23.6; 28.7; 28.8 and 31.6 ($(\underline{C}H_2)_5CH_{3\text{ isomer 1 and 2}}$); 29.60 ($\underline{C}H_2CHN_{\text{isomer 1}}$); 29.65 ($\underline{C}H_2CHN_{\text{isomer 2}}$); 40.2 ($\underline{C}H_2C{=}O_{\text{isomer 1}}$); 40.3 ($\underline{C}H_2C{=}O_{\text{isomer 2}}$); 47.5 ($\underline{C}HBr_{\text{isomer 1}}$); 48.0 ($\underline{C}HBr_{\text{isomer 2}}$); 49.83 ($\underline{C}HN_{\text{isomer 1}}$); 49.88 ($\underline{C}HN_{\text{isomer 2}}$); 66.0 ($\underline{C}H_2O_{\text{isomer 1 and 2}}$); 164.9 ($N\underline{C}{=}O_{\text{isomer 1}}$); 165.1 ($N\underline{C}{=}O_{\text{isomer 2}}$); 174.2 ($O\underline{C}{=}O_{\text{isomer 1}}$); 174.3 ($O\underline{C}{=}O_{\text{isomer 2}}$); 200.3 ($CH_2\underline{C}{=}O_{\text{isomer 1}}$); 200.5 ($CH_2\underline{C}{=}O_{\text{isomer 2}}$). **MS (ESI):** *m/z* (%): 348/350 ($M + H^+$, 30).

HRMS mass calculated: $C_{14}H_{22}BrNO_4H^+$: 348.0810; **obtained:** 348.0795. **IR (cm^{-1}) ν_{max}:** 1018; 1176 (C-O); 1543 (HN-C=O); 1648 (HN-C=O); 1727 ($C{=}O_{ketone}$); 1771 ($C{=}O_{lactone}$); 2850 (CH); 2928 (CH); 2953 (CH); 3284 (NH). **Melting Point:** 155 °C. White powder. **Y:** 63%.

S1.6. N*-(2-Bromo-3-oxododecanoyl)-(*S*)-homoserine Lactone* **2f**

S1.6.1. (IUPAC: 2-Bromo-3-oxo-*N*-[(3*S*)-tetrahydro-2-oxo-3-furanyl]-dodecanamide)

^{1}H NMR (300 MHz, $CDCl_3$): δ 0.88 (3H, t, *J* = 7.2Hz, $C\underline{H}_{3\text{ isomer 1 and 2}}$); 1.23–1.33 (12H, m, $(C\underline{H}_2)_6CH_{3\text{ isomer 1 and 2}}$); 1.52–1.69 (2H, m, $C\underline{H}_2(CH_2)_6CH_{3\text{ isomer 1 and 2}}$); 2.25 (1H, dddd, *J* = 11.1 Hz, 11.1 Hz, 11.1 Hz, 9.1 Hz, $OCH_2C\underline{H}H'_{\text{isomer 1 and 2}}$); 2.75 (2H, t, *J* = 7.2 Hz, $C\underline{H}_2C{=}O_{\text{isomer 1 and 2}}$); 2.79–2.89 (1H, m, $OCH_2CH\underline{H}'_{\text{isomer 1 and 2}}$); 4.30 (1H, ddd, *J* = 11.0 Hz, 9.4 Hz, 6.1 Hz, $OC\underline{H}H'_{\text{isomer 1 and 2}}$); 4.51 (1H, t, *J* = 9.0 Hz, $OCH\underline{H}'_{\text{isomer 1 and 2}}$); 4.52-4.60 (1H, m, $C\underline{H}N_{\text{isomer 1 and 2}}$); 4.77 (0.5H, s, $C\underline{H}Br_{\text{isomer 1}}$); 4.80 (0.5H, s, $C\underline{H}Br_{\text{isomer 2}}$); 7.16 (1H, s, $N\underline{H}_{\text{isomer 1 and 2}}$).

^{13}C NMR (75 MHz, $CDCl_3$): δ 14.1 ($\underline{C}H_{3\text{ isomer 1 and 2}}$); 22.7; 23.6; 28.8; 29.2; 29.3; 29.4 and 31.8 ($(\underline{C}H_2)_7CH_{3\text{ isomer 1 and 2}}$); 29.65 ($\underline{C}H_2CHN_{\text{isomer 1}}$); 29.70 ($\underline{C}H_2CHN_{\text{isomer 2}}$); 40.3 ($\underline{C}H_2C{=}O_{\text{isomer 1}}$); 40.4 ($\underline{C}H_2C{=}O_{\text{isomer 2}}$); 47.4 ($\underline{C}HBr_{\text{isomer 1}}$); 47.9 ($\underline{C}HBr_{\text{isomer 2}}$); 49.84 ($\underline{C}HN_{\text{isomer 1}}$); 49.90 ($\underline{C}HN_{\text{isomer 2}}$); 66.0 ($\underline{C}H_2O_{\text{isomer 1 and 2}}$); 164.9 ($N\underline{C}{=}O_{\text{isomer 1}}$); 165.1 ($N\underline{C}{=}O_{\text{isomer 2}}$); 174.2 ($O\underline{C}{=}O_{\text{isomer 1}}$); 174.3 ($O\underline{C}{=}O_{\text{isomer 2}}$); 200.3 ($CH_2\underline{C}{=}O_{\text{isomer 1}}$); 200.5 ($CH_2\underline{C}{=}O_{\text{isomer 2}}$). **MS (ESI):** *m/z* (%): 376/378 ($M + H^+$, 30).

HRMS mass calculated: $C_{16}H_{26}BrNO_4H^+$: 376.1123; **obtained:** 376.1104. **IR (cm^{-1}) ν_{max}:** 1018; 1174 (C-O); 1545 (HN-C=O); 1652 (HN-C=O); 1730 ($C{=}O_{ketone}$); 1773 ($C{=}O_{lactone}$); 2853 (CH); 2928 (CH); 2954 (CH); 3285 (NH). **Melting Point:** 155 °C. White powder. **Y:** 57%.

S1.7. N*-(2,2-Dibromo-3-oxohexanoyl)-(*S*)-homoserine Lactone* **3a**

S1.7.1. (IUPAC: 2,2-Dibromo-3-oxo-*N*-[(3*S*)-tetrahydro-2-oxo-3-furanyl]-hexanamide)

^{1}H NMR (300 MHz, $CDCl_3$): δ 0.94 (3H, t, *J* = 7.2 Hz, $C\underline{H}_3$); 1.69 (2H, sext, *J* = 7.2 Hz, $C\underline{H}_2CH_3$); 2.23 (1H, dddd, *J* = 11.7 Hz, 11.7 Hz, 11.7 Hz, 8.8 Hz, $OCH_2C\underline{H}H'$); 2.86 (1H, dddd, *J* = 13.1 Hz, 8.9 Hz, 6.2 Hz, 1.3 Hz, $OCH_2CH\underline{H}'$); 2.92 (2H, t, *J* = 7.2 Hz, $C\underline{H}_2C{=}O$); 4.30 (1H, ddd, *J* = 11.0 Hz, 9.4 Hz, 6.1 Hz, $OC\underline{H}H'$); 4.54 (1H, td, *J* = 9.4 Hz, 1.1 Hz, $OCH\underline{H}'$); 4.58 (1H, ddd, *J* = 11.4 Hz, 8.7 Hz, 6.2 Hz, $C\underline{H}N$); 7.38 (1H, d, *J* = 6.1 Hz, $N\underline{H}$).

^{13}C NMR (75 MHz, $CDCl_3$): δ 13.4 ($\underline{C}H_3$); 18.4 ($\underline{C}H_2CH_3$); 29.4 ($\underline{C}H_2CHN$); 38.2 ($\underline{C}H_2C{=}O$); 50.6 ($\underline{C}HN$); 61.3 ($\underline{C}Br_2$); 66.2 ($\underline{C}H_2O$); 164.5 ($N\underline{C}{=}O$); 174.2 ($O\underline{C}{=}O$); 194.2 ($CH_2\underline{C}{=}O$). **MS (ESI):** *m/z* (%): 369/371/373 ($M + H^+$, 59/100/49).

HRMS mass calculated: $C_{10}H_{13}Br_2NO_4H^+$: 369.9290; **obtained:** 369.9281. **IR (cm^{-1}) ν_{max}:** 1041; 1164 (C-O); 1523 (HN-C=O); 1674 (HN-C=O); 1741 ($C{=}O_{ketone}$); 1774 ($C{=}O_{lactone}$); 2871 (CH); 2926 (CH); 2947 (CH); 3352 (NH). **$[\alpha]^{25}_D$:** −19.8 (c 1.8; CH_2Cl_2). Yellow oil. **Y:** 67%.

S1.8. N*-(2,2-Dibromo-3-oxoheptanoyl)-(*S*)-homoserine Lactone* **3b**

S1.8.1. (IUPAC: 2,2-Dibromo-3-oxo-*N*-[(3*S*)-tetrahydro-2-oxo-3-furanyl]-heptanamide)

^{1}H NMR (300 MHz, $CDCl_3$): δ 0.93 (3H, t, *J* = 7.2 Hz, $C\underline{H}_3$); 1.37 (2H, sext, *J* = 7.2 Hz, $C\underline{H}_2CH_3$); 1.68 (2H, quint, *J* = 7.2 Hz, $C\underline{H}_2CH_2CH_3$); 2.35 (1H, dddd, *J* = 11.7 Hz, 11.7 Hz, 11.7 Hz, 8.8 Hz, $OCH_2C\underline{H}H'$); 2.83 (1H, dddd, *J* = 13.1 Hz, 8.9 Hz, 6.2 Hz, 1.1 Hz, $OCH_2CH\underline{H}'$); 2.92 (2H, t, *J* = 7.2 Hz, $C\underline{H}_2C{=}O$); 4.34 (1H, ddd, *J* = 11.0 Hz, 9.4 Hz, 6.1 Hz, $OC\underline{H}H'$); 4.53 (1H, td, *J* = 9.4 Hz, 1.1 Hz, $OCH\underline{H}'$); 4.59 (1H, ddd, *J* = 11.4 Hz, 8.7 HZ, 6.2 Hz, $C\underline{H}N$); 7.57 (1H, d, *J* = 6.1 Hz, $N\underline{H}$).

^{13}C NMR (75 MHz, $CDCl_3$): δ 13.8 ($\underline{C}H_3$); 22.0 ($\underline{C}H_2CH_3$); 27.1 ($\underline{C}H_2CH_2CH_3$); 28.5 ($\underline{C}H_2CHN$); 36.1 ($\underline{C}H_2C{=}O$); 50.4 ($\underline{C}HN$); 61.9 ($\underline{C}Br_2$); 66.2 ($\underline{C}H_2O$); 164.5 ($N\underline{C}{=}O$); 174.2 ($O\underline{C}{=}O$); 194.2 ($CH_2\underline{C}{=}O$). **MS (ESI):** *m/z* (%): 401/403/405 ($M + NH_4^+$, 59/100/49).

HRMS mass calculated: $C_{11}H_{15}Br_2NO_4H^+$: 383.9446; **obtained:** 383.9441. **IR (cm^{-1}) ν_{max}:** 1021; 1176 (C-O); 1518 (HN-C=O); 1671 (HN-C=O); 1741 ($C{=}O_{ketone}$); 1774 ($C{=}O_{lactone}$); 2872 (CH); 2931 (CH); 2959 (CH); 3349 (NH). **$[\alpha]^{25}_D$:** −26.2 (c 1.5; CH_2Cl_2). Yellow oil. **Y:** 66%.

S1.9. N*-(2,2-Dibromo-3-oxooctanoyl)-(*S*)-homoserine Lactone* **3c**

S1.9.1. (IUPAC: 2,2-Dibromo-3-oxo-*N*-[(3*S*)-tetrahydro-2-oxo-3-furanyl]-octanamide)

^{1}H NMR (300 MHz, $CDCl_3$): δ 0.94 (3H, t, *J* = 7.2 Hz, $C\underline{H}_3$); 1.24–1.36 (4H, m, $(C\underline{H}_2)_2CH_3$); 1.69 (2H, sext, *J* = 7.2 Hz, $C\underline{H}_2(CH_2)_2CH_3$); 2.23 (1H, dddd, *J* = 11.7 Hz, 11.7 Hz, 11.7 Hz, 8.8 Hz, $OCH_2C\underline{H}H'$); 2.86 (1H, dddd, *J* = 13.1 Hz, 8.9 Hz, 6.2 Hz, 1.3 Hz, $OCH_2CH\underline{H}'$); 2.92 (2H, t, *J* = 7.2 Hz, $C\underline{H}_2C{=}O$); 4.30 (1H, ddd, *J* = 11.0 Hz, 9.4 Hz, 6.1 Hz, $OC\underline{H}H'$); 4.54 (1H, td, *J* = 9.4 Hz, 1.1 Hz, $OCH\underline{H}'$); 4.58 (1H, ddd, *J* = 11.4 Hz, 8.7 Hz, 6.2 Hz, $C\underline{H}N$); 7.38 (1H, d, *J* = 6.1 Hz, $N\underline{H}$).

^{13}C NMR (75 MHz, $CDCl_3$): δ 13.4 ($\underline{C}H_3$); 18.4; 22.3 and 31.0 ($(\underline{C}H_2)_3CH_3$); 29.4 ($\underline{C}H_2CHN$); 38.2 ($\underline{C}H_2C{=}O$); 50.6 ($\underline{C}HN$); 61.3 ($\underline{C}Br_2$); 66.2 ($\underline{C}H_2O$); 164.5 ($N\underline{C}{=}O$); 174.2 ($O\underline{C}{=}O$); 194.2 ($CH_2\underline{C}{=}O$). **MS (ESI):** *m/z* (%): 397/399/401 ($M + H^+$, 59/100/49).

HRMS mass calculated: $C_{12}H_{17}Br_2NO_4H^+$: 397.9603; **obtained:** 397.9589. **IR (cm^{-1}) ν_{max}:** 1027; 1169 (C-O); 1518 (HN-C=O); 1670 (HN-C=O); 1743 ($C{=}O_{ketone}$); 1774 ($C{=}O_{lactone}$); 2862 (CH); 2933 (CH); 2959 (CH); 3349 (NH). **$[\alpha]_D^{25}$:** −24.8 (c 3.1; CH_2Cl_2). Yellow oil. **Y:** 64%.

S1.10. N-*(2,2-Dibromo-3-oxononanoyl)-(*S*)-homoserine Lactone* **3d**

S1.10.1. (IUPAC: 2,2-Dibromo-3-oxo-*N*-[(3*S*)-tetrahydro-2-oxo-3-furanyl]-nonanamide)

^{1}H NMR (300 MHz, $CDCl_3$): δ 0.89 (3H, t, *J* = 7.2 Hz, $C\underline{H}_3$); 1.22–1.42 (6H, m, $(C\underline{H}_2)_3CH_3$); 1.69 (2H, quint, *J* = 7.2 Hz, $C\underline{H}_2(CH_2)_3CH_3$); 2.31 (1H, dddd, *J* = 11.7 Hz, 11.7 Hz, 11.7 Hz, 8.8 Hz, $OCH_2C\underline{H}H'$); 2.84–2.93 (1H, m, $OCH_2CH\underline{H}'$); 2.92 (2H, t, *J* = 7.2 Hz, $C\underline{H}_2C{=}O$); 4.34 (1H, ddd, *J* = 11.0 Hz, 9.4 Hz, 6.1 Hz, $OC\underline{H}H'$); 4.54 (1H, td, *J* = 9.4 Hz, 1.1 Hz, $OCH\underline{H}'$); 4.56 (1H, ddd, *J* = 11.4 Hz, 8.8 Hz, 6.1 Hz, $C\underline{H}N$); 7.45 (1H, d, *J* = 6.1 Hz, $N\underline{H}$).

^{13}C NMR (75 MHz, $CDCl_3$): δ 14.1 ($\underline{C}H_3$); 22.5 ($\underline{C}H_2CH_3$); 25.0; 29.4 and 31.5 ($(\underline{C}H_2)_3CH_2CH_3$); 28.6 ($\underline{C}H_2CHN$); 36.5 ($\underline{C}H_2C{=}O$); 50.6 ($\underline{C}HN$); 61.5 ($\underline{C}Br_2$); 66.2 ($\underline{C}H_2O$); 164.5 ($N\underline{C}{=}O$); 174.0 ($O\underline{C}{=}O$); 194.3 ($CH_2\underline{C}{=}O$). **MS (ESI):** *m/z* (%): 429/431/433 ($M + NH_4^+$, 100).

HRMS mass calculated: $C_{13}H_{19}Br_2NO_4H^+$: 413.9738; **obtained:** 413.9734. **IR (cm^{-1}) ν_{max}:** 1023; 1174 (C-O); 1509 (HN-C=O); 1676 (HN-C=O); 1741 ($C{=}O_{ketone}$); 1774 ($C{=}O_{lactone}$); 2852 (CH); 2867 (CH); 2928 (CH); 3345 (NH). **$[\alpha]_D^{25}$:** −29.1 (c 1.2; CH_2Cl_2). **Melting point:** 88 °C. White powder. **Y:** 62%.

S1.11. N-*(2,2-Dibromo-3-oxodecanoyl)-(*S*)-homoserine Lactone* **3e**

S1.11.1. (IUPAC: 2,2-Dibromo-3-oxo-*N*-[(3*S*)-tetrahydro-2-oxo-3-furanyl]-decanamide)

^{1}H NMR (300 MHz, $CDCl_3$): δ 0.88 (3H, t, *J* = 7.2 Hz, $C\underline{H}_3$); 1.18-1.39 (8H, m, $(C\underline{H}_2)_4CH_3$); 1.69 (2H, quint, *J* = 7.1 Hz, $C\underline{H}_2(CH_2)_4CH_3$); 2.23–2.41 (1H, m, $OCH_2C\underline{H}H'$); 2.81–2.90 (1H, m, $OCH_2CH\underline{H}'$); 2.92 (2H, t, *J* = 7.1 Hz, $C\underline{H}_2C{=}O$); 4.28–4.40 (1H, m, $OC\underline{H}H'$); 4.53 (1H, m, $C\underline{H}N$); 4.49–4.62 (1H, m, $OCH\underline{H}'$); 7.49 (1H, d, *J* = 6.1 Hz, $N\underline{H}$).

^{13}C NMR (75 MHz, $CDCl_3$): δ 14.2 ($\underline{C}H_3$); 22.7 ($\underline{C}H_2CH_3$); 25.0; 28.9; 29.3 and 31.7 ($(\underline{C}H_2)_4CH_2CH_3$); 29.0 ($\underline{C}H_2CHN$); 36.5 ($\underline{C}H_2C{=}O$); 50.6 ($\underline{C}HN$); 61.6 ($\underline{C}Br_2$); 66.2 ($\underline{C}H_2O$); 164.5 ($N\underline{C}{=}O$); 174.0 ($O\underline{C}{=}O$); 194.3 ($CH_2\underline{C}{=}O$). **MS (ESI):** *m/z* (%): 424/426/428 ($M + H^+$, 100).

HRMS mass calculated: $C_{14}H_{21}Br_2NO_4H^+$: 425.9910; **obtained:** 425.9909. **IR (cm^{-1}) ν_{max}:** 1023; 1174 (C-O); 1509 (HN-C=O); 1646 (HN-C=O); 1739 ($C{=}O_{ketone}$); 1773 ($C{=}O_{lactone}$); 2852 (CH); 2868 (CH); 2924 (CH); 3337 (NH). **$[\alpha]_D^{25}$:** +3.0 (c 1.2; CH_2Cl_2). **Melting point:** 68 °C. White powder. **Y:** 71%.

S1.12. N-*(2,2-Dibromo-3-oxododecanoyl)-(*S*)-homoserine Lactone* **3f**

S1.12.1. (IUPAC: 2,2-Dibromo-3-oxo-*N*-[(3*S*)-tetrahydro-2-oxo-3-furanyl]-dodecanamide)

^{1}H NMR (300 MHz, CDCl$_3$): δ 0.88 (3H, t, *J* = 7.2 Hz, C<u>H</u>$_3$); 1.04–1.17 (2H, m, C<u>H</u>$_2$CH$_2$CH$_3$); 1.18–1.42 (10H, m, CH$_2$C<u>H</u>$_2$CH$_3$ and (C<u>H</u>$_2$)$_5$CH$_2$CH$_2$H$_3$); 1.69 (2H, quint, *J* = 7.2 Hz, C<u>H</u>$_2$(CH$_2$)$_6$CH$_3$); 2.24–2.40 (1H, m, OCH$_2$C<u>H</u>H'); 2.82–2.94 (1H, m, OCH$_2$CH<u>H</u>'); 2.91 (2H, t, *J* = 7.2 Hz, C<u>H</u>$_2$C=O); 4.28–4.39 (1H, m, OC<u>H</u>H'); 4.49–4.61 (2H, m, C<u>H</u>N and OCH<u>H</u>'); 7.54 (1H, d, *J* = 6.1 Hz, N<u>H</u>).

^{13}C NMR (75 MHz, CDCl$_3$): δ 14.2 (<u>C</u>H$_3$); 22.7 (<u>C</u>H$_2$CH$_3$); 25.0; 28.9; 29.3; 29.4; 29.5 and 31.9 ((<u>C</u>H$_2$)$_6$CH$_2$CH$_3$); 29.2 (<u>C</u>H$_2$CHN); 36.5 (<u>C</u>H$_2$C=O); 50.6 (<u>C</u>HN); 66.2 (<u>C</u>H$_2$O); 82.3 (<u>C</u>Br$_2$); 164.5 (N<u>C</u>=O); 174.0 (O<u>C</u>=O); 194.3 (CH$_2$<u>C</u>=O). **MS (ESI):** *m/z* (%): 454/454/456 (M + H$^+$, 100).

HRMS mass calculated: C$_{16}$H$_{25}$Br$_2$NO$_4$NH$_4^+$: 471.0489 M + NH$_4^+$; **obtained:** 471.0486 M + NH$_4^+$**. IR (cm^{-1}) ν$_{max}$:** 1023; 1174 (C-O); 1509 (HN-C=O); 1646 (HN-C=O); 1739 (C=O$_{ketone}$); 1773 (C=O$_{lactone}$); 2852 (CH); 2868 (CH); 2924 (CH); 3337 (NH). **$[\alpha]_D^{25}$:** +4.9 (c 1.6; CH$_2$Cl$_2$)**. Melting point:** 70 °C. White powder. **Y:** 68%.

S1.13. N-*(2,2-Dibromoacetyl)-(*S*)-homoserine Lactone* **5**

S1.13.1. (IUPAC: 2,2-Dibromo-*N*-[(3*S*)-tetrahydro-2-oxo-3-furanyl]-acetamide)

^{1}H NMR (300 MHz, CDCl$_3$): δ 2.24 (1H, dddd, *J* = 12.1 Hz, 12.1 Hz, 12.1 Hz, 11.9 Hz, 8.8 Hz, OCH$_2$C<u>H</u>H'); 2.92 (1H, dddd, *J* = 12.7 Hz, 8.8 Hz, 6.1 Hz, 1.1 Hz, OCH$_2$CH<u>H</u>'); 4.33 (1H, ddd, *J* = 11.6 Hz, 8.6 Hz, 6.1 Hz, OC<u>H</u>H'); 4.49–4.57 (2H, m, OCH<u>H</u>' and C<u>H</u>N); 5.85 (1H, s, C<u>H</u>Br$_2$); 6.99 (1H, s, N<u>H</u>).

^{13}C NMR (75 MHz, CDCl$_3$): δ 30.4 (<u>C</u>H$_2$CHN); 50.1 (<u>C</u>HN); 50.7 (<u>C</u>HBr$_2$); 66.2 (<u>C</u>H$_2$O); 165.0 (N<u>C</u>=O); 174.1 (O<u>C</u>=O). **MS (ESI):** *m/z* (%): 298/300/302 (M + H$^+$).

HRMS mass calculated: C$_6$H$_7$Br$_2$NO$_3^-$: 209.97302; **obtained:** 209.9729. **IR (cm^{-1}) ν$_{max}$:** 1166 (C-O); 1552 (HN-C=O); 1664 (HN-C=O); 1774 (C=O$_{lactone}$); 3026 (CH); 3280 (NH). **Melting Point:** 184 °C. White powder. **Y:** 38%.

S1.14. N-*(2-Iodo-3-oxohexanoyl)-(*S*)-homoserine Lactone* **10a**

S1.14.1. (IUPAC: 2-Iodo-3-oxo-*N*-[(3*S*)-tetrahydro-2-oxo-3-furanyl]-hexanamide)

^{1}H NMR (300 MHz, CDCl$_3$): δ 0.94 (3H, t, *J* = 7.2 Hz, C<u>H</u>$_{3\ isomer\ 1\ and\ 2}$); 1.67 (2H, sext, *J* = 7.2 Hz, C<u>H</u>$_2$CH$_{3\ isomer\ 1\ and\ 2}$); 2.29 (1H, dddd, *J* = 11.2 Hz, 11.2 Hz, 11.0 Hz, 8.9 Hz, OCH$_2$C<u>H</u>H'$_{isomer\ 1\ and\ 2}$); 2.73 (2H, t, *J* = 7.2 Hz, C<u>H</u>$_2$C=O$_{isomer\ 1\ and\ 2}$); 2.75–2.89 (1H, m, OCH$_2$CH<u>H</u>'$_{isomer\ 1\ and\ 2}$); 4.30 (1H, ddd, *J* = 11.0 Hz, 9.4 Hz, 6.1 Hz, OC<u>H</u>H'$_{isomer\ 1\ and\ 2}$); 4.50 (1H, t, *J* = 9.0 Hz, OCH<u>H</u>'$_{isomer\ 1\ and\ 2}$); 4.58–4.67 (1H, m, C<u>H</u>N$_{isomer\ 1\ and\ 2}$); 5.01 (1H, s, C<u>H</u>I$_{isomer\ 1}$); 7.80 (0.5 H, d, *J* = 6.1 Hz, N<u>H</u>$_{isomer\ 1}$); 7.90 (0.5 H, d, *J* = 6.1 Hz, N<u>H</u>$_{isomer\ 2}$).

^{13}C NMR (75 MHz, CDCl$_3$): **δ** 13.3 ($\underline{C}H_{3\text{ isomer 1 and 2}}$); 17.3 ($\underline{C}H_2CH_{3\text{ isomer 1 and 2}}$); 20.2 ($\underline{C}HI_{\text{isomer 1}}$); 21.0 ($\underline{C}HI_{\text{isomer 2}}$); 28.9 ($\underline{C}H_2CHN_{\text{isomer 1}}$); 29.3 ($\underline{C}H_2CHN_{\text{isomer 2}}$); 42.10 ($\underline{C}H_2C{=}O_{\text{isomer 1}}$); 42.17 ($\underline{C}H_2C{=}O_{\text{isomer 2}}$); 49.5 ($\underline{C}HN_{\text{isomer 1}}$); 49.7 ($\underline{C}HN_{\text{isomer 2}}$); 65.9 ($\underline{C}H_2O_{\text{isomer 1 and 2}}$); 167.0 ($N\underline{C}{=}O_{\text{isomer 1}}$); 167.4 ($N\underline{C}{=}O_{\text{isomer 2}}$); 174.4 ($O\underline{C}{=}O_{\text{isomer 1}}$); 174.5 ($O\underline{C}{=}O_{\text{isomer 2}}$); 203.6 ($CH_2\underline{C}{=}O_{\text{isomer 1}}$); 203.9 ($CH_2\underline{C}{=}O_{\text{isomer 2}}$). **MS (ESI):** *m/z* (%): 340 (M + H^+, 30).

HRMS mass calculated: $C_{10}H_{14}INO_4H^+$: 340.0046; **obtained:** 340.0034. **IR (cm^{-1}) ν$_{max}$:** 1018; 1174 (C-O); 1546 (HN-C=O); 1652 (HN-C=O); 1730 ($C{=}O_{ketone}$); 1774 ($C{=}O_{lactone}$); 2878 (CH); 2964 (CH); 3070 (CH); 3287 (NH). **Melting Point:** 159 °C. Yellowish powder. **Y:** 68%.

S1.15. N*-(2-Iodo-3-oxoheptanoyl)-(*S*)-homoserine Lactone* **10b**

S1.15.1. (IUPAC: 2-Iodo-3-oxo-*N*-[(3*S*)-tetrahydro-2-oxo-3-furanyl]-heptanamide)

^{1}H NMR (300 MHz, CDCl$_3$): **δ** 0.93 (3H, t, *J* = 7.3 Hz, $C\underline{H}_{3\text{ isomer 1 and 2}}$); 1.34 (2H, sext, *J* = 7.2 Hz, $C\underline{H}_2CH_{3\text{ isomer 1 and 2}}$); 1.63 (2H, m, $C\underline{H}_2CH_2CH_{3\text{ isomer 1 and 2}}$); 2.29 (1H, dddd, *J* = 11.1 Hz, 11.1 Hz, 11.1 Hz, 9.2 Hz, $OCH_2C\underline{H}H'_{\text{isomer 1 and 2}}$); 2.67–2.83 (2H, m, $C\underline{H}_2C{=}O_{\text{isomer 1 and 2}}$); 2.84–2.95 (1H, m, $OCH_2CH\underline{H}'_{\text{isomer 1 and 2}}$); 4.30 (1H, ddd, *J* = 11.0 Hz, 9.4 Hz, 6.1 Hz, $OC\underline{H}H'_{\text{isomer 1 and 2}}$); 4.50 (1H, t, *J* = 9.2 Hz, $OCH\underline{H}'_{\text{isomer 1 and 2}}$); 4.58-4.66 (1H, m, $C\underline{H}N_{\text{isomer 1 and 2}}$); 5.01 (1H, s, $C\underline{H}I_{\text{isomer 1 and 2}}$); 7.80 (0.5H, d, *J* = 6.1 Hz, $N\underline{H}_{\text{isomer 1}}$); 7.90 (0.5H, d, *J* = 6.1 Hz, $N\underline{H}_{\text{isomer 2}}$).

^{13}C NMR (75 MHz, CDCl$_3$): **δ** 13.8 ($\underline{C}H_{3\text{ isomer 1 and 2}}$); 20.2 ($\underline{C}HI_{\text{isomer 1}}$); 21.0 ($\underline{C}HI_{\text{isomer 2}}$); 21.9 ($\underline{C}H_2CH_{3\text{ isomer 1 and 2}}$); 25.7 ($\underline{C}H_2CH_2CH_{3\text{ isomer 1 and 2}}$); 28.9 ($\underline{C}H_2CHN_{\text{isomer 1}}$); 29.2 ($\underline{C}H_2CHN_{\text{isomer 2}}$); 40.04 ($\underline{C}H_2C{=}O_{\text{isomer 1}}$); 40.09 ($\underline{C}H_2C{=}O_{\text{isomer 2}}$); 49.5 ($\underline{C}HN_{\text{isomer 1}}$); 49.7 ($\underline{C}HN_{\text{isomer 2}}$); 65.9 ($\underline{C}H_2O_{\text{isomer 1 and 2}}$); 167.0 ($N\underline{C}{=}O_{\text{isomer 1}}$); 167.4 ($N\underline{C}{=}O_{\text{isomer 2}}$); 174.39 ($O\underline{C}{=}O_{\text{isomer 1}}$); 174.44 ($O\underline{C}{=}O_{\text{isomer 2}}$); 203.8 ($CH_2\underline{C}{=}O_{\text{isomer 1}}$); 204.1 ($CH_2\underline{C}{=}O_{\text{isomer 2}}$). **MS (ESI):** *m/z* (%): 354 (M + H^+, 30).

HRMS mass calculated: $C_{11}H_{16}INO_4H^+$: 354.0202; **obtained:** 354.0196. **IR (cm^{-1}) ν$_{max}$:** 1018; 1179 (C-O); 1540 (HN-C=O); 1642 (HN-C=O); 1719 ($C{=}O_{ketone}$); 1772 ($C{=}O_{lactone}$); 2872 (CH); 2938 (CH); 2956 (CH); 3293 (NH). **Melting Point:** 156 °C. Yellowish powder. **Y:** 63%.

S1.16. N*-(2-Iodo-3-oxooctanoyl)-(*S*)-homoserine Lactone* **10c**

S1.16.1. (IUPAC: 2-Iodo-3-oxo-*N*-[(3*S*)-tetrahydro-2-oxo-3-furanyl]-octanamide)

^{1}H NMR (300 MHz, CDCl$_3$): **δ** 0.93 (3H, t, *J* = 7.1 Hz, $C\underline{H}_{3\text{ isomer 1 and 2}}$); 1.23–1.35 (4H, m, $(C\underline{H}_2)_2CH_{3\text{ isomer 1 and 2}}$); 1.63 (2H, quint, *J* = 7.2 Hz, $C\underline{H}_2(CH_2)_2CH_{3\text{ isomer 1 and 2}}$); 2.28 (1H, m, $OCH_2C\underline{H}H'_{\text{isomer 1 and 2}}$); 2.75 (2H, t, *J* = 7.2 Hz, $C\underline{H}_2C{=}O_{\text{isomer 1 and 2}}$); 2.79–2.86 (1H, m, $OCH_2CH\underline{H}'_{\text{isomer 1 and 2}}$); 4.31 (1H, ddd, *J* = 11.0 Hz, 9.4 Hz, 6.1 Hz, $OC\underline{H}H'_{\text{isomer 1 and 2}}$); 4.51 (1H, t, *J* = 9.0 Hz, $OCH\underline{H}'_{\text{isomer 1 and 2}}$); 4.52–4.60 (1H, m, $C\underline{H}N_{\text{isomer 1 and 2}}$); 5.0 (1H, s, $C\underline{H}I_{\text{isomer 1 and 2}}$); 7.9 (1H, d, *J* = 6.1 Hz, $N\underline{H}_{\text{isomer 1 and 2}}$).

^{13}C NMR (75 MHz, CDCl$_3$): **δ** 13.9 ($\underline{C}H_{3\text{ isomer 1 and 2}}$); 19.5 ($\underline{C}HI_{\text{isomer 1}}$); 20.1 ($\underline{C}HI_{\text{isomer 2}}$); 22.6 and 31.8 ($(\underline{C}H_2)_2CH_{3\text{ isomer 1 and 2}}$); 23.8 ($\underline{C}H_2CH_2C{=}O_{\text{isomer 1 and 2}}$); 29.60 ($\underline{C}H_2CHN_{\text{isomer 1}}$); 29.70 ($\underline{C}H_2CHN_{\text{isomer 2}}$); 40.1 ($\underline{C}H_2C{=}O_{\text{isomer 1}}$); 40.3 ($\underline{C}H_2C{=}O_{\text{isomer 2}}$); 49.6 ($\underline{C}HN_{\text{isomer 1}}$); 49.8 ($\underline{C}HN_{\text{isomer 2}}$);

66.0 ($\underline{C}H_2O_{isomer\ 1\ and\ 2}$); 164.8 ($N\underline{C}{=}O_{isomer\ 1}$); 165.0 ($N\underline{C}{=}O_{isomer\ 2}$); 174.1 ($O\underline{C}{=}O_{isomer\ 1}$); 174.2 ($O\underline{C}{=}O_{isomer\ 2}$); 200.3 ($CH_2\underline{C}{=}O_{isomer\ 1}$); 200.5 ($CH_2\underline{C}{=}O_{isomer\ 2}$). **MS (ESI):** *m/z* (%): 369 ($M + H^+$, 30).

HRMS mass calculated: $C_{12}H_{18}INO_4H^+$: 368.0359; **obtained:** 368.0348. **IR (cm^{-1}) ν_{max}:** 1021; 1178 (C-O); 1548 (HN-C=O); 1651 (HN-C=O); 1732 ($C{=}O_{ketone}$); 1774 ($C{=}O_{lactone}$); 2892 (CH); 2960 (CH); 3073 (CH); 3276 (NH). **Melting Point:** 158 ℃. Yellowish powder. **Y:** 61%.

S1.17. N-*(2-Iodo-3-oxononanoyl)-(*S*)-homoserine Lactone* **10d**

S1.17.1. (IUPAC: 2-Iodo-3-oxo-*N*-[(3*S*)-tetrahydro-2-oxo-3-furanyl]-nonanamide)

^{1}H NMR (300 MHz, $CDCl_3$): δ 0.94 (3H, t, *J* = 7.2 Hz, $C\underline{H}_{3\ isomer\ 1\ and\ 2}$); 1.20–1.39 (6H, m, $(C\underline{H}_2)_3CH_{3\ isomer\ 1\ and\ 2}$); 1.63 (2H, quint, *J* = 7.2 Hz, $C\underline{H}_2(CH_2)_3CH_{3\ isomer\ 1\ and\ 2}$); 2.27 (1H, dddd, *J* = 11.8 Hz, 11.7 Hz, 11.7 Hz, 9.1 Hz, $OCH_2C\underline{H}H'_{isomer\ 1\ and\ 2}$); 2.75 (2H, t, *J* = 7.2 Hz, $C\underline{H}_2C{=}O_{isomer\ 1\ and\ 2}$); 2.76–2.91 (1H, m, $OCH_2CH\underline{H}'_{isomer\ 1\ and\ 2}$); 4.30 (1H, ddd, *J* = 11.0 Hz, 9.4 Hz 6.1 Hz, $OC\underline{H}H'_{isomer\ 1\ and\ 2}$); 4.51 (1H, t, *J* = 9.0 Hz, $OCH\underline{H}'_{isomer\ 1\ and\ 2}$); 4.53–4.60 (1H, m, $C\underline{H}N_{isomer\ 1\ and\ 2}$); 4.79 (0.5H, s, $C\underline{H}I_{isomer\ 1}$); 4.82 (0.5H, s, $C\underline{H}I_{isomer\ 2}$); 7.27 (0.5H, d, *J* = 6.1 Hz, $N\underline{H}_{isomer\ 1}$); 7.34 (0.5H, d, *J* = 6.1 Hz, $N\underline{H}_{isomer\ 2}$).

^{13}C NMR (75 MHz, $CDCl_3$): δ 14.1 ($\underline{C}H_{3\ isomer\ 1\ and\ 2}$); 20.2 ($\underline{C}HI_{isomer\ 1}$); 21.0 ($\underline{C}HI_{isomer\ 2}$); 22.5; 28.6 and 31.5 ($(\underline{C}H_2)_3CH_{3\ isomer\ 1\ and\ 2}$); 23.7 ($\underline{C}H_2CH_2C{=}O_{isomer\ 1\ and\ 2}$); 29.62 ($\underline{C}H_2CHN_{isomer\ 1}$); 29.64 ($\underline{C}H_2CHN_{isomer\ 2}$); 40.3 ($\underline{C}H_2C{=}O_{isomer\ 1}$); 40.4 ($\underline{C}H_2C{=}O_{isomer\ 2}$); 49.88 ($\underline{C}HN_{isomer\ 1}$); 49.94 ($\underline{C}HN_{isomer\ 2}$); 66.1 ($\underline{C}H_2O_{isomer\ 1\ and\ 2}$); 165.0 ($N\underline{C}{=}O_{isomer\ 1}$); 165.1 ($N\underline{C}{=}O_{isomer\ 2}$); 174.0 ($O\underline{C}{=}O_{isomer\ 1}$); 174.1 ($O\underline{C}{=}O_{isomer\ 2}$); 200.5 ($CH_2\underline{C}{=}O_{isomer\ 1}$); 200.7 ($CH_2\underline{C}{=}O_{isomer\ 2}$). **MS (ESI):** *m/z* (%): 382 ($M + H^+$, 30).

HRMS mass calculated: $C_{13}H_{20}INO_4H^+$: 382.0515; **obtained:** 382.0506. **IR (cm^{-1}) ν_{max}:** 1018; 1177 (C-O); 1542 (HN-C=O); 1642 (HN-C=O); 1729 ($C{=}O_{ketone}$); 1773 ($C{=}O_{lactone}$); 2859 (CH); 2934 (CH); 2932 (CH); 3292 (NH). **Melting Point:** 156 ℃. Yellowish powder. **Y:** 59%.

S1.18. N-*(2-Iodo-3-oxodecanoyl)-(*S*)-homoserine Lactone* **10e**

S1.18.1. (IUPAC: 2-Iodo-3-oxo-*N*-[(3*S*)-tetrahydro-2-oxo-3-furanyl]-decanamide)

^{1}H NMR (300 MHz, $CDCl_3$): δ 0.92 (3H, t, *J* = 7.2 Hz, $C\underline{H}_{3\ isomer\ 1\ and\ 2}$); 1.24–1.33 (8H, m, $(C\underline{H}_2)_4CH_{3\ isomer\ 1\ and\ 2}$); 1.58-1.66 (2H, m, $C\underline{H}_2(CH_2)_4CH_{3\ isomer\ 1\ and\ 2}$); 2.25 (1H, dddd, *J* = 11.1 Hz, 11.1 Hz, 11.1 Hz, 9.1 Hz, $OCH_2C\underline{H}H'_{isomer\ 1\ and\ 2}$); 2.75 (2H, t, *J* = 7.2 Hz, $C\underline{H}_2C{=}O_{isomer\ 1\ and\ 2}$); 2.79–2.89 (1H, m, $OCH_2CH\underline{H}'_{isomer\ 1\ and\ 2}$); 4.31 (1H, ddd, *J* = 11.0 Hz, 9.4 Hz, 6.1 Hz, $OC\underline{H}H'_{isomer\ 1\ and\ 2}$); 4.51 (1H, t, *J* = 9.0 Hz, $OCH\underline{H}'_{isomer\ 1\ and\ 2}$); 4.52–4.60 (1H, m, $C\underline{H}N_{isomer\ 1\ and\ 2}$); 4.77 (0.5H, s, $C\underline{H}I_{isomer\ 1}$); 4.80 (0.5H, s, $C\underline{H}I_{isomer\ 2}$); 7.20 (0.5H, d, *J* = 6.1 Hz, $N\underline{H}_{isomer\ 1}$); 7.21 (0.5H, d, *J* = 6.1 Hz, $N\underline{H}_{isomer\ 2}$).

^{13}C NMR (75 MHz, $CDCl_3$): δ 14.4 ($\underline{C}H_{3\ isomer\ 1\ and\ 2}$); 22.6; 28.7; 28.8 and 31.6 ($(\underline{C}H_2)_4CH_{3\ isomer\ 1\ and\ 2}$); 23.6 ($\underline{C}H_2CH_2C{=}O_{isomer\ 1\ and\ 2}$); 29.60 ($\underline{C}H_2CHN_{isomer\ 1}$); 29.65 ($\underline{C}H_2CHN_{isomer\ 2}$); 40.2 ($\underline{C}H_2C{=}O_{isomer\ 1}$); 40.3 ($\underline{C}H_2C{=}O_{isomer\ 2}$); 47.5 ($\underline{C}HI_{isomer\ 1}$); 48.0 ($\underline{C}HI_{isomer\ 2}$); 49.83 ($\underline{C}HN_{isomer\ 1}$); 49.88 ($\underline{C}HN_{isomer\ 2}$); 66.0 ($\underline{C}H_2O_{isomer\ 1\ and\ 2}$); 164.9 ($N\underline{C}{=}O_{isomer\ 1}$); 165.1

($N\underline{C}{=}O_{isomer\ 2}$); 174.2 ($O\underline{C}{=}O_{isomer\ 1}$); 174.3 ($O\underline{C}{=}O_{isomer\ 2}$); 200.3 ($CH_2\underline{C}{=}O_{isomer\ 1}$); 200.5 ($CH_2\underline{C}{=}O_{isomer\ 2}$). **MS (ESI):** *m/z* (%): 396 (M + H^+, 30).

HRMS mass calculated: $C_{14}H_{22}INO_4H^+$: 396.0672; **obtained:** 396.0659. **IR (cm^{-1}) ν_{max}:** 1018; 1174 (C-O); 1545 (HN-C=O); 1652 (HN-C=O); 1730 ($C{=}O_{ketone}$); 1773 ($C{=}O_{lactone}$); 2853 (CH); 2928 (CH); 2954 (CH); 3285 (NH). **Melting Point:** 152 ℃. Yellowish powder. **Y:** 63%.

S1.19. N-*(2-Iodo-3-oxododecanoyl)-(*S*)-homoserine Lactone* **10f**

S1.19.1. (IUPAC: 2-Iodo-3-oxo-*N*-[(3*S*)-tetrahydro-2-oxo-3-furanyl]-dodecanamide)

^{1}H NMR (300 MHz, $CDCl_3$): δ 0.93 (3H, t, *J* = 7.0 Hz, $C\underline{H}_{3\ isomer\ 1\ and\ 2}$); 1.23–1.33 (12H, m, $(C\underline{H}_2)_6CH_{3\ isomer\ 1\ and\ 2}$); 1.52-1.69 (2H, m, $C\underline{H}_2(CH_2)_6CH_{3\ isomer\ 1\ and\ 2}$); 2.25 (1H, dddd, *J* = 11.1 Hz, 11.1 Hz, 11.1 Hz, 9.1 Hz, $OCH_2C\underline{H}H'_{isomer\ 1\ and\ 2}$); 2.75 (2H, t, *J* = 7.0 Hz, $C\underline{H}_2C{=}O_{isomer\ 1\ and\ 2}$); 2.79–2.89 (1H, m, $OCH_2CH\underline{H}'_{isomer\ 1\ and\ 2}$); 4.30 (1H, ddd, *J* = 11.0 Hz, 9.4 Hz, 6.1 Hz, $OC\underline{H}H'_{isomer\ 1\ and\ 2}$); 4.51 (1H, t, *J* = 9.1 Hz, $OCH\underline{H}'_{isomer\ 1\ and\ 2}$); 4.52–4.60 (1H, m, $C\underline{H}N_{isomer\ 1\ and\ 2}$); 4.77 (0.5H, s, $C\underline{H}I_{isomer\ 1}$); 4.80 (0.5H, s, $C\underline{HI}_{isomer\ 2}$); 7.16 (1H, s, $N\underline{H}_{isomer\ 1\ and\ 2}$).

^{13}C NMR (75 MHz, $CDCl_3$): δ 14.1 ($\underline{C}H_{3\ isomer\ 1\ and\ 2}$); 20.1 ($\underline{C}HI_{isomer\ 1}$); 20.2 ($\underline{C}HI_{isomer\ 2}$); 22.7; 28.8; 29.2; 29.3; 29.4 and 31.8 ($(\underline{C}H_2)_6CH_{3\ isomer\ 1\ and\ 2}$); 23.6 ($\underline{C}H_2CH_2C{=}O_{isomer\ 1\ and\ 2}$); 29.65 ($\underline{C}H_2CHN_{isomer\ 1}$); 29.70 ($\underline{C}H_2CHN_{isomer\ 2}$); 40.3 ($\underline{C}H_2C{=}O_{isomer\ 1}$); 40.4 ($\underline{C}H_2C{=}O_{isomer\ 2}$); 49.84 ($\underline{C}HN_{isomer\ 1}$); 49.90 ($\underline{C}HN_{isomer\ 2}$); 66.0 ($\underline{C}H_2O_{isomer\ 1\ and\ 2}$); 164.9 ($N\underline{C}{=}O_{isomer\ 1}$); 165.1 ($N\underline{C}{=}O_{isomer\ 2}$); 174.2 ($O\underline{C}{=}O_{isomer\ 1}$); 174.3 ($O\underline{C}{=}O_{isomer\ 2}$); 200.3 ($CH_2\underline{C}{=}O_{isomer\ 1}$); 200.5 ($CH_2\underline{C}{=}O_{isomer\ 2}$). **MS (ESI):** *m/z* (%): 424 (M + H^+, 30).

HRMS mass calculated: $C_{16}H_{26}INO_4H^+$: 424.0985; **obtained:** 424.0978. **IR (cm^{-1}) ν_{max}:** 1016; 1178 (C-O); 1545 (HN-C=O); 1652 (HN-C=O); 1716 ($C{=}O_{ketone}$); 1772 ($C{=}O_{lactone}$); 2850 (CH); 2928 (CH); 2954 (CH); 3290 (NH). **Melting Point:** 153 ℃. Yellowish powder. **Y:** 57%.

S1.20. N-*(2,2-Dichloro-3-oxohexanoyl)-(*S*)-homoserine Lactone* **11a**

S1.20.1. (IUPAC: 2,2-Dichloro-3-oxo-*N*-[(3*S*)-tetrahydro-2-oxo-3-furanyl]-hexanamide)

^{1}H NMR (300 MHz, $CDCl_3$): δ 0.94 (3H, t, *J* = 7.2 Hz, $C\underline{H}_3$); 1.70 (2H, quint, *J* = 7.2 Hz, $C\underline{H}_2CH_3$); 2.23–2.35 (1H, m, $OCH_2C\underline{H}H'$); 2.82–2.92 (3H, m, $OCH_2CH\underline{H}'$ and $C\underline{H}_2C{=}O$); 4.30–4.37 (1H, m, $OC\underline{H}H'$); 4.52 (1H, td, *J* = 9.0 Hz, 1.0 Hz, $OCH\underline{H}'$); 4.56 (1H, m, $C\underline{H}N$); 7.30 (1H, d, *J* = 6.1 Hz, $N\underline{H}$).

^{13}C NMR (75 MHz, $CDCl_3$): δ 13.3 ($\underline{C}H_3$); 17.9 ($\underline{C}H_2CH_3$); 29.4 ($\underline{C}H_2CHN$); 38.1 ($\underline{C}H_2C{=}O$); 50.3 ($\underline{C}HN$); 66.1 ($\underline{C}H_2O$); 82.2 ($\underline{C}Cl_2$); 163.9 ($N\underline{C}{=}O$); 173.8 ($O\underline{C}{=}O$); 194.4 ($CH_2\underline{C}{=}O$). **MS (ESI):** *m/z* (%): 299/301 (M + NH_4^+, 100).

HRMS mass calculated: $C_{10}H_{13}Cl_2NO_4H^+$: 282.0300; **obtained:** 282.0286. **IR (cm^{-1}) ν_{max}:** 1023; 1174 (C-O); 1509 (HN-C=O); 1646 (HN-C=O); 1739 ($C{=}O_{ketone}$); 1773 ($C{=}O_{lactone}$); 2852 (CH); 2868 (CH); 2924 (CH); 3337 (NH). **$[\alpha]_D^{25}$:** −3.2 (c 1.2; CH_2Cl_2). Colorless oil. **Y:** 71%.

S1.21. N-*(2,2-Dichloro-3-oxoheptanoyl)-(*S*)-homoserine Lactone* **11b**

S1.21.1. (IUPAC: 2,2-Dichloro-3-oxo-*N*-[(3*S*)-tetrahydro-2-oxo-3-furanyl]-heptanamide)

^{1}H NMR (300 MHz, $CDCl_3$): **δ** 0.92 (3H, t, *J* = 7.2 Hz, $C\underline{H}_3$); 1.36 (2H, sext, *J* = 7.2 Hz, $C\underline{H}_2CH_3$); 1.66 (2H, quint, *J* = 7.2 Hz, $C\underline{H}_2CH_2CH_3$); 2.37 (1H, dddd, *J* = 11.6 Hz, 11.6 Hz, 11.6 Hz, 8.8 Hz, $OCH_2C\underline{H}H'$); 2.75–2.81 (1H, m, $OCH_2CH\underline{H}'$); 2.84 (2H, t, *J* = 7.2 Hz, $C\underline{H}_2C{=}O$); 4.34 (1H, ddd, *J* = 11.0 Hz, 9.4 Hz, 6.1 Hz, $OC\underline{H}H'$); 4.52 (1H, td, *J* = 9.4 Hz, 1.1 Hz, $OCH\underline{H}'$); 4.63 (1H, ddd, *J* = 11.6 Hz, 8.8 Hz, 6.6 Hz, $C\underline{H}N$); 7.61 (1H, d, *J* = 6.1 Hz, $N\underline{H}$).

^{13}C NMR (75 MHz, $CDCl_3$): **δ** 13.8 ($\underline{C}H_3$); 22.0 ($\underline{C}H_2CH_3$); 26.3 ($\underline{C}H_2CH_2CH_3$); 28.9 ($\underline{C}H_2CHN$); 36.1 ($\underline{C}H_2C{=}O$); 50.2 ($\underline{C}HN$); 66.2 ($\underline{C}H_2O$); 82.5 ($\underline{C}Cl_2$); 164.0 ($N\underline{C}{=}O$); 174.2 ($O\underline{C}{=}O$); 194.6 ($CH_2\underline{C}{=}O$). **MS (ESI):** *m/z* (%): 313/315 (M + NH_4^+, 100).

HRMS mass calculated: $C_{11}H_{15}Cl_2NO_4H^+$: 296.0456; **obtained:** 296.0449. **IR (cm^{-1}) ν_{max}:** 1022; 1178 (C-O); 1522 (HN-C=O); 1680 (HN-C=O); 1750 ($C{=}O_{ketone}$); 1776 ($C{=}O_{lactone}$); 2873 (CH); 2934 (CH); 2961 (CH); 3347 (NH). **$[\alpha]_D^{25}$:** −13.2 (c 4.5; CH_2Cl_2). Yellow oil. **Y:** 69%.

S1.22. N-*(2,2-Dichloro-3-oxooctanoyl)-(*S*)-homoserine Lactone* **11c**

S1.22.1. (IUPAC: 2,2-Dichloro-3-oxo-*N*-[(3*S*)-tetrahydro-2-oxo-3-furanyl]-octanamide)

^{1}H NMR (300 MHz, $CDCl_3$): **δ** 0.89 (3H, t, *J* = 7.2 Hz, $C\underline{H}_3$); 1.27–1.38 (4H, m, $(C\underline{H}_2)_2CH_3$); 1.68 (2H, quint, *J* = 7.1 Hz, $C\underline{H}_2(CH_2)_2CH_3$); 2.23–2.35 (1H, m, $OCH_2C\underline{H}H'$); 2.82-2.92 (3H, m, $OCH_2CH\underline{H}'$ and $C\underline{H}_2C{=}O$); 4.30–4.37 (1H, m, $OC\underline{H}H'$); 4.52 (1H, td, *J* = 9.0 Hz, 1.0 Hz, $C\underline{H}N$); 4.56 (1H, m, $OCH\underline{H}'$); 7.32 (1H, d, *J* = 6.1 Hz, $N\underline{H}$).

^{13}C NMR (75 MHz, $CDCl_3$): **δ** 13.8 ($\underline{C}H_3$); 22.3 and 30.9 ($(\underline{C}H_2)_2CH_3$); 23.8 ($\underline{C}H_2(CH_2)_2CH_3$); 28.8 ($\underline{C}H_2CHN$); 36.3 ($\underline{C}H_2C{=}O$); 50.3 ($\underline{C}HN$); 66.1 ($\underline{C}H_2O$); 82.2 ($\underline{C}Cl_2$); 163.9 ($N\underline{C}{=}O$); 173.8 ($O\underline{C}{=}O$); 194.6 ($CH_2\underline{C}{=}O$). **MS (ESI):** *m/z* (%): 327/329 (M + NH_4^+, 100).

HRMS mass calculated: $C_{12}H_{17}Cl_2NO_4H^+$: 310.0613; **obtained:** 310.0602. **IR (cm^{-1}) ν_{max}:** 1023; 1174 (C-O); 1509 (HN-C=O); 1646 (HN-C=O); 1739 ($C{=}O_{ketone}$); 1773 ($C{=}O_{lactone}$); 2852 (CH); 2868 (CH); 2924 (CH); 3337 (NH). **$[\alpha]_D^{25}$:** +3.0 (c 4.9; CH_2Cl_2). Colorless oil. **Y:** 74%.

S1.23. N-*(2,2-Dichloro-3-oxononanoyl)-(*S*)-homoserine Lactone* **11d**

S1.23.1. (IUPAC: 2,2-Dichloro-3-oxo-*N*-[(3*S*)-tetrahydro-2-oxo-3-furanyl]-nonanamide)

^{1}H NMR (300 MHz, $CDCl_3$): **δ** 0.88 (3H, t, *J* = 7.2Hz, $C\underline{H}_3$); 1.23–1.37 (6H, m, $(C\underline{H}_2)_3CH_3$); 1.66 (2H, quint, *J* = 7.2 Hz, $C\underline{H}_2(CH_2)_3CH_3$); 2.38 (1H, dddd, *J* = 11.7 Hz, 11.7 Hz, 11.6 Hz, 8.8 Hz, $OCH_2C\underline{H}H'$); 2.72–2.81 (1H, m, $OCH_2CH\underline{H}'$); 2.83 (2H, t, *J* = 7.2 Hz, $C\underline{H}_2C{=}O$); 4.34 (1H, ddd, *J* = 10.5 Hz, 9.4 Hz, 6.1 Hz, $OC\underline{H}H'$); 4.51 (1H, t, *J* = 8.8 Hz, $OCH\underline{H}'$); 4.63 (1H, ddd, *J* = 11.6 Hz, 8.8 Hz, 6.6 Hz, $C\underline{H}N$); 7.69 (1H, d, *J* = 6.6 Hz, $N\underline{H}$).

^{13}C NMR (75 MHz, $CDCl_3$): **δ** 14.1 (<u>C</u>H_3); 22.5; 28.8 and 31.5 ((<u>C</u>H_2)$_3$$CH_3$); 24.3 (<u>C</u>$H_2$($CH_2$)$_3$$CH_3$); 28.7 (<u>C</u>$H_2$CHN); 36.4 (<u>C</u>$H_2$C=O); 50.2 (<u>C</u>HN); 66.2 (<u>C</u>H_2O); 82.5 (<u>C</u>Cl_2); 164.0 (N<u>C</u>=O); 174.3 (O<u>C</u>=O); 194.6 (CH_2<u>C</u>=O). **MS (ESI):** *m/z* (%): 341/343 (M + NH_4^+, 100).

HRMS mass calculated: $C_{13}H_{19}Cl_2NO_4H^+$: 324.0769; **obtained:** 324.0763. **IR (cm^{-1}) ν_{max}:** 1019; 1176 (C-O); 1523 (HN-C=O); 1682 (HN-C=O); 1779 (C=$O_{lactone}$); 2858 (CH); 2867 (CH); 2928 (CH); 3334 (NH). **$[\alpha]^{25}_{D}$:** −10.1 (c 6.1; CH_2Cl_2). Yellow oil. **Y:** 68%.

S1.24. N-*(2,2-Dichloro-3-oxodecanoyl)-(*S*)-homoserine Lactone* **11e**

S1.24.1. (IUPAC: 2,2-Dichloro-3-oxo-*N*-[(3*S*)-tetrahydro-2-oxo-3-furanyl]-decanamide)

^{1}H NMR (300 MHz, $CDCl_3$): **δ** 0.88 (3H, t, *J* = 7.2 Hz, C<u>H</u>$_3$); 1.25–1.34 (8H, m, (C<u>H</u>$_2$)$_4$$CH_3$); 1.67 (2H, quint, *J* = 7.1 Hz, C<u>H</u>$_2$(CH_2)$_4$$CH_3$); 2.23–2.41 (1H, m, O$CH_2$C<u>H</u>H'); 2.81–2.92 (3H, m, OCH_2C<u>H</u>H' and C<u>H</u>$_2$C=O); 4.28–4.40 (1H, m, OC<u>H</u>H'); 4.50 (1H, td, *J* = 9.0 Hz, 1.0 Hz, C<u>H</u>N); 4.55 (1H, m, OCH<u>H</u>'); 7.32 (1H, d, *J* = 6.1 Hz, N<u>H</u>).

^{13}C NMR (75 MHz, $CDCl_3$): **δ** 14.2 (<u>C</u>H_3); 22.5; 25.0; 28.9; 29.3 and 31.7 ((<u>C</u>H_2)$_5$$CH_3$); 28.8 (<u>C</u>$H_2$CHN); 36.4 (<u>C</u>$H_2$C=O); 50.3 (<u>C</u>HN); 61.6 (<u>C</u>Cl_2); 66.2 (<u>C</u>H_2O); 164.5 (N<u>C</u>=O); 174.0 (O<u>C</u>=O); 194.3 (CH_2<u>C</u>=O). **MS (ESI):** *m/z* (%): 355/357 (M + NH_4^+, 100). **HRMS mass calculated:** $C_{14}H_{21}Cl_2NO_4H^+$: 338.0926; **obtained:** 338.0914. **IR (cm^{-1}) ν_{max}:** 1019; 1182 (C-O); 1509 (HN-C=O); 1646 (HN-C=O); 1748 (C=O_{ketone}); 1774 (C=$O_{lactone}$); 2857 (CH); 2868 (CH); 2954 (CH); 3319 (NH). **$[\alpha]^{25}_{D}$:** −14.0 (c 3.1; CH_2Cl_2). Colorless oil.**Y:** 73%.

S1.25. N-*(2,2-Dichloro-3-oxododecanoyl)-(*S*)-homoserine Lactone* **11f**

S1.25.1. (IUPAC: 2,2-Dichloro-3-oxo-*N*-[(3*S*)-tetrahydro-2-oxo-3-furanyl]-dodecanamide)

^{1}H NMR (300 MHz, $CDCl_3$): **δ** 0.88 (3H, t, *J* = 7.1 Hz, C<u>H</u>$_3$); 1.22–1.36 (12H, m, (C<u>H</u>$_2$)$_6$$CH_3$); 1.67 (2H, quint, *J* = 7.1 Hz, C<u>H</u>$_2$(CH_2)$_6$$CH_3$); 2.29 (1H, dddd, *J* = 11.7 Hz, 11.7 Hz, 11.6 Hz, 8.8 Hz, OCH_2C<u>H</u>H'); 2.84 (2H, t, *J* = 7.2 Hz, C<u>H</u>$_2$C=O); 2.87–2.94 (1H, m, OCH_2CH<u>H</u>'); 4.28–4.40 (1H, m, OC<u>H</u>H'); 4.50 (1H, td, *J* = 9.0 Hz, 1.0 Hz, OCH<u>H</u>'); 4.55 (1H, m, C<u>H</u>N); 7.32 (1H, d, *J* = 6.1 Hz, N<u>H</u>).

^{13}C NMR (75 MHz, $CDCl_3$): **δ** 14.2 (<u>C</u>H_3); 22.5; 24.3; 25.0; 28.9; 29.3; 31.7 and 32.4 ((<u>C</u>H_2)$_6$$CH_3$); 28.8 (<u>C</u>$H_2$CHN); 36.4 (<u>C</u>$H_2$C=O); 50.3 (<u>C</u>HN); 61.6 (<u>C</u>Cl_2); 66.2 (<u>C</u>H_2O); 164.5 (N<u>C</u>=O); 174.0 (O<u>C</u>=O); 194.3 (CH_2<u>C</u>=O). **MS (ESI):** *m/z* (%): 424/426/428 (M + H^+, 100).

HRMS mass calculated: $C_{16}H_{25}Cl_2NO_4H^+$: 366.1239; **obtained:** 366.1216. **IR (cm^{-1}) ν_{max}:** 1023; 1174 (C-O); 1509 (HN-C=O); 1646 (HN-C=O); 1739 (C=O_{ketone}); 1773 (C=$O_{lactone}$); 2852 (CH); 2868 (CH); 2924 (CH); 3337 (NH). **$[\alpha]^{25}_{D}$:** +3.0 (c 2.2; CH_2Cl_2). Yellow oil. **Y:** 73%.

S1.26. N-*(2,2-Dichloroacetyl)-(*S*)-homoserine Lactone* **15**

S1.26.1. (IUPAC: 2,2-Dichloro-*N*-[(3*S*)-tetrahydro-2-oxo-3-furanyl]-acetamide)

^{1}H NMR (300 MHz, $CDCl_3$): δ 2.25 (1H, dddd, *J* = 12.1 Hz, 12.1 Hz, 12.1 Hz, 11.9 Hz, 8.8 Hz, $OCH_2C\underline{H}H$'); 2.92 (1H, dddd, *J* = 12.7 Hz, 8.8 Hz, 6.1 Hz, 1.1 Hz, $CH_2CH\underline{H}$'); 4.34 (1H, ddd, *J* = 11.6 Hz, 8.6 Hz, 6.1 Hz, $OC\underline{H}H$'); 4.53 (1H, t, *J* = 8.8 Hz, $OCH\underline{H}$'); 4.54–4.59 (1H, m, $C\underline{H}N$); 5.98 (1H, s, $C\underline{H}Cl_2$); 7.03 (1H, s, $N\underline{H}$).

^{13}C NMR (75 MHz, $CDCl_3$): δ 29.9 ($\underline{C}H_2CHN$); 50.0 ($\underline{C}HN$); 65.7 ($\underline{C}HCl_2$); 66.2 ($\underline{C}H_2O$); 164.8 ($N\underline{C}$=O); 174.1 ($O\underline{C}$=O). **MS (ESI):** *m/z* (%): 229/231/233 (M + NH_4^+).

HRMS mass calculated: $C_6H_7Cl_2NO_3^-$: 209.97302; **obtained:** 209.9729. **IR (cm^{-1}) ν_{max}:** 1192 (C-O); 1557 (HN-C=O); 1672 (HN-C=O); 1766 ($C=O_{lactone}$); 2924 (CH); 3284 (NH). **Melting Point:** 163 °C. White powder. **Y:** 41%.

© 2014 by the authors; licensee MDPI, Basel, Switzerland. This article is an open access article distributed under the terms and conditions of the Creative Commons Attribution license (http://creativecommons.org/licenses/by/3.0/).
